# Selective Metal Chelation by a Thiosemicarbazone Derivative Interferes with Mitochondrial Respiration and Ribosome Biogenesis in Candida albicans

**DOI:** 10.1128/spectrum.01951-21

**Published:** 2022-04-18

**Authors:** Ximeng Duan, Zhiyu Xie, Liying Ma, Xueyang Jin, Ming Zhang, Yuliang Xu, Yue Liu, Hongxiang Lou, Wenqiang Chang

**Affiliations:** a Department of Natural Product Chemistry, Key Laboratory of Chemical Biology (Ministry of Education), School of Pharmaceutical Sciences, Cheeloo College of Medicine, Shandong University, Jinan, Shandong Province, China; b Key Laboratory of Micro-Nano Materials for Energy Storage and Conversion of Henan Province, Institute of Surface Micro and Nano Materials, College of Chemical and Materials Engineering, Xuchang University, Xuchang, Henan, People’s Republic of China; c State Key Laboratory of Esophageal Cancer Prevention and Treatment, Key Laboratory of Technology of Drug Preparation (Zhengzhou University), Ministry of Education of China, Key Laboratory of Henan Province for Drug Quality and Evaluation, Institute of Pharmaceutical Research and School of Pharmaceutical Sciences, Zhengzhou University, Zhengzhou, China; d Institute of Medical Science, The Second Hospital, Cheeloo College of Medicine, Shandong University, Jinan, Shandong, China; University of Minnesota Medical School

**Keywords:** thiosemicarbazone derivatives, antifungal, metal chelator, mitochondrial respiration, ribosome biogenesis

## Abstract

Metal chelation is generally considered as a promising antifungal approach but its specific mechanisms are unclear. Here, we identify 13 thiosemicarbazone derivatives that exert broad-spectrum antifungal activity with potency comparable or superior to that of fluconazole *in vitro* by screening a small compound library comprising 89 thiosemicarbazone derivatives as iron chelators. Among the hits, 19ak exhibits minimal cytotoxicity and potent activity against either azole-sensitive or azole-resistant fungal pathogens. Mechanism investigations reveal that 19ak inhibits mitochondrial respiration mainly by retarding mitochondrial respiratory chain complex I activity through iron chelation, and further reduces mitochondrial membrane potential and ATP synthesis in Candida albicans. In addition, 19ak inhibits fungal ribosome biogenesis mainly by disrupting intracellular zinc homeostasis. 19ak also stimulates the activities of antioxidant enzymes and decreases reactive oxygen species formation in C. albicans, resulting in an increase in detrimental intracellular reductive stress. However, 19ak has minor effects on mammalian cells in depleting intracellular iron and zinc. Moreover, 19ak exhibits low capacity to induce drug resistance and *in vivo* efficacy in a Galleria mellonella infection model. These findings uncover retarded fungal mitochondrial respiration and ribosome biogenesis as downstream effects of disruption of iron and zinc homeostasis in C. albicans and provide a basis for the thiosemicarbazone 19ak in antifungal application.

**IMPORTANCE** The increasing incidence of fungal infections and resistance to existing antifungals call for the development of broad-spectrum antifungals with novel mechanisms of action. In this study, we demonstrate that a thiosemicarbazone derivative 19ak selectively inhibits mitochondrial respiration mainly by retarding mitochondrial respiratory chain complex I activity through iron chelation and inhibits ribosome biogenesis mainly by disrupting intracellular zinc homeostasis in C. albicans. In addition, 19ak exhibits low capacity to induce fungal resistance, minimal cytotoxicity, and *in vivo* antifungal efficacy. This study provides the basis of thiosemicarbazone derivative 19ak as a metal chelator for the treatment of fungal infections.

## INTRODUCTION

Invasive fungal infections are a leading cause of morbidity and mortality in immunocompromised individuals, and the increase in the immunocompromised patient population in recent decades has been accompanied by a rise in the incidence of clinically relevant infections (1). It is estimated that more than 300 million people worldwide are adversely affected by fungal-related infections each year, directly or indirectly causing more than 1.6 million deaths (2). In the United States alone, the estimated annual medical cost for treating fungal diseases surpasses 7.2 billion dollars (3). Despite the substantial societal and financial burdens posed by fungal infections, few antifungals are available, particularly for the treatment of invasive fungal infections. Currently used antifungal drugs mainly fall into three major classes: polyenes, azoles, and echinocandins. While echinocandins have a great safety profile, other conventional antifungals, such as amphotericin B, voriconazole (VOR), and itraconazole, possess severe toxicity (4, 5). The difficulty of treating fungal infections is further enhanced by the development of resistance to traditional antifungal drugs due to their widespread use.

To overcome these challenges, there is an urgent need to identify effective antifungal agents with new mechanisms of action. The host can hinder microbial growth and virulence through restricting the access of microbes to key micronutrients such as iron and zinc, which is termed as nutritional immunity (6). This suggests that preventing metal acquisition by fungal pathogens through metal chelation is an alternative approach to copy with fungal infections. Iron is the most abundant trace metal and an essential nutrient for eukaryotic cells (7, 8). Because of its redox properties, it plays a pivotal role in many core metabolic pathways, such as cell respiration, electron transport chains, iron-sulfur cluster assembly, and oxygen transfer (9). In most fungal pathogens, mitochondrial respiratory chain complex I (CI) is the largest enzyme complex containing iron-sulfur centers (10). Mutants of the subunits of CI exhibit deficiencies in respiration and fungal pathogenesis (11). The important role of iron implies that iron chelators may interfere with iron metabolism and impact fungal respiration to function as antifungals. For example, the iron chelator ciclopirox olamine (CPX) potently inhibits fungal cell growth (12). In addition to antifungal activity, some iron-chelating agents increase the sensitivity of fungi to traditional antifungal drugs such as fluconazole (FLC) and nystatin (13).

Zinc is another important micronutrient and participates in various transduction signaling pathways. The major zinc-binding proteins in fungal species include Cu^2+^/Zn^2+^ superoxide dismutases (SODs), alcohol dehydrogenase, and ribosomal proteins (14), which are involved in regulating fungal cell growth and pathogenesis. Therefore, zinc has been considered to be essential for fungal growth and pathogenicity (15, 16). For example, disruption of zinc transporter Zrt1 in C. albicans results in growth defects in zinc-restricted medium (17). A variety of zinc chelators have been demonstrated to exhibit antifungal activities or potentiate the efficacy of present antifungal drugs (18–21).

As iron chelators, thiosemicarbazone derivatives have played an important role in organic and medicinal chemistry due to their promising biological activities, which include antiparasitic (22), anticancer (23, 24), anticonvulsant (25), antiviral (26), antimicrobial (27, 28), and antifungal (29) effects. For example, 3-aminopyridine-2-carboxaldehyde thiosemicarbazone (Triapine), which exhibits anti-tumor activity, has entered phase II clinical trials and is expected to be used to treat many different types of cancer (30), and 4-(pyridine-2-yl)-N-([(8E)-5,6,7,8-tetrahydroquinolin-8-ylidene]amino) piperazine-1-carbothioamide (COTI-2) has been evaluated in phase Ib/IIa clinical trials for the treatment of gynecologic malignancies (31). In addition to iron, thiosemicarbazone derivatives chelate zinc and reactivate mutant p53 by restoring zinc binding to zinc-deficient p53 mutants for tumor chemotherapy and radiation sensitization (32). In previous work, our collaborators investigated the antitumor activity of 87 thiosemicarbazone derivatives or their ability to reverse meropenem resistance in New Delhi metallo-β-lactamase-1-positive bacteria (28, 33). However, little is known about their antifungal activities and potential mechanisms.

In this study, we systematically evaluated the antifungal activities of these thiosemicarbazone derivatives. Several exhibited broad and potent activity against three common fungal genera: *Candida*, Cryptococcus, and Aspergillus. Compound 19ak, which had the most potent activity against *Candida* species and minimal cytotoxicity against mammalian cells, was chosen for further investigation. 19ak reduced intracellular iron and zinc levels and further inhibited mitochondrial respiration and ribosome biogenesis in C. albicans. The inhibition of mitochondrial respiration resulted in a decrease in mitochondrial membrane potential (mt*Δψ*) and intracellular ATP and reactive oxygen species (ROS) generation, leading to an increase in detrimental reductive stress. However, we did not observe notable depletion of ferrous and zinc ion by 19ak in our tested mammalian cells and the respiratory activity of mammalian cells was also not distinctly affected. 19ak exhibited low capacity to induce drug resistance, probably due to a lack of energy to support resistance mechanisms in treated cells. Finally, 19ak significantly improved the survival of C. albicans-infected Galleria mellonella. These findings support further potential applications of thiosemicarbazone derivative 19ak in treating fungal infections.

## RESULTS

### Thiosemicarbazone derivatives are potent broad-spectrum antifungal agents.

A total of 87 thiosemicarbazones from our collaborators and two commercial analogues were screened for antifungal activity. The structures and activities of these derivatives are summarized in Table 1 and Table S1. We first assessed the antifungal activity of the 89 thiosemicarbazones against C. albicans SC5314, a wild-type strain commonly used for antifungal evaluation. Thirteen hits possessed potent activity against C. albicans SC5314 with MIC values ranging from 0.125 to 2 μg/mL, comparable or superior to that of the antifungal drug FLC (Table 1). To further evaluate the antifungal activities of these 13 hits, we utilized another two representative strains, Cryptococcus neoformans H99 and Aspergillus fumigatus AF293. Among these hits, 19ak, 19t, and 19k exhibited potent antifungal activities against all three fungal species (Table 1). We then investigated the *in vitro* activities of 19ak, 19t, and 19k against additional fungal strains, including a panel of azole-resistant strains with a variety of resistance mechanisms. The results showed that 19ak, 19t, and 19k were highly active against all tested C. albicans strains and one representative strain of Candida krusei, Candida parapsilosis, Candida tropicalis, C. neoformans, and A. fumigatus, with MICs ranging from 0.125 to 4 μg/mL (Table 2). In addition, we evaluated the minimal fungicidal concentration (MFC). The MFC value of 19ak was 512 times higher than its MIC value, and similar results were obtained for 19t and 19k (Table S2), suggesting that these thiosemicarbazone derivatives act as fungistatic agents.

**TABLE 1:**
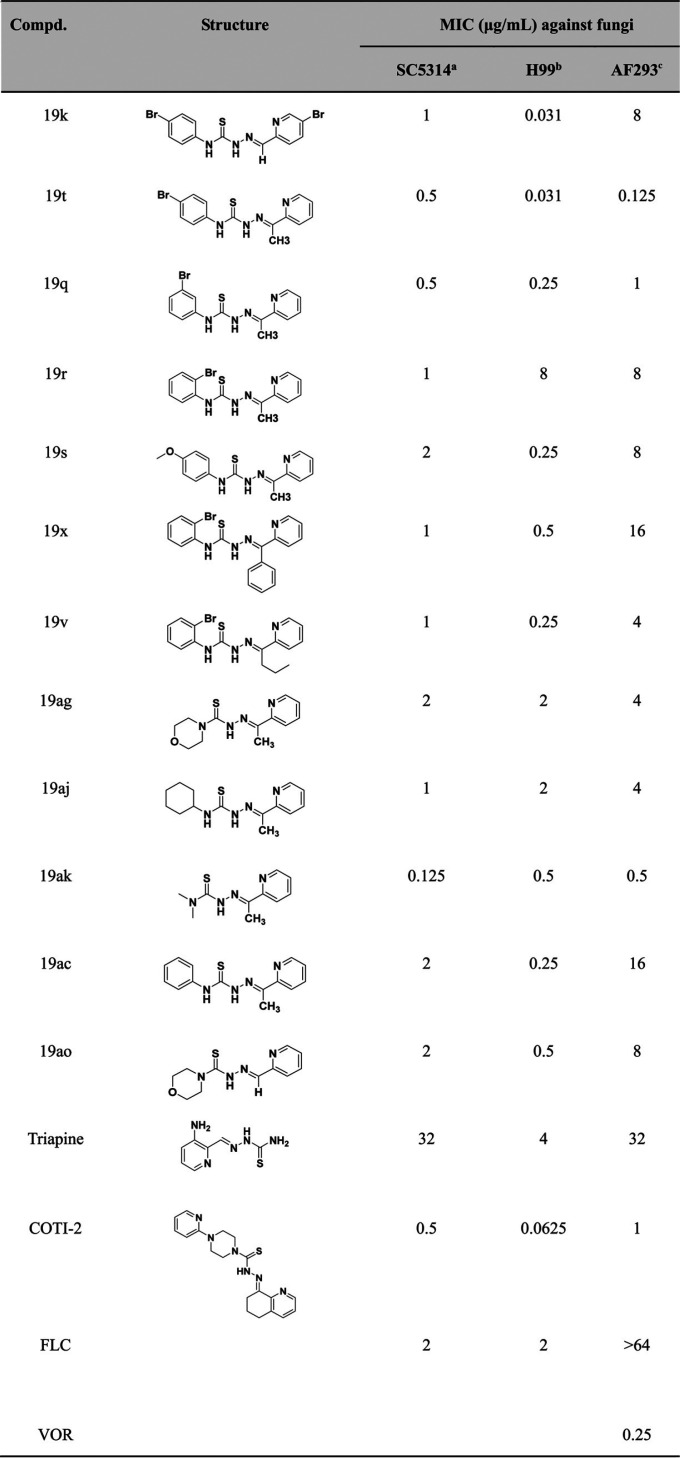
MIC values (μg/mL) of the hits against pathogenic fungi

a SC5314 is a C. albicans wild-type strain.

b H99 is a C. neoformans wild-type strain.

c AF293 is a standard strain of A. fumigatus.

**TABLE 2:** MICs of 19ak, 19t, and 19k against individual fungal strains

Species	Strains	MIC_80_ (μg/mL)				
		19ak	19t	19k	FLC	VOR^*a*^
C. albicans	DSY654	0.125	0.25	1	0.25	nd
C. albicans	YEM13	0.5	1	2	64	nd
C. albicans	YEM15	0.25	1	2	64	nd
C. albicans	G5	1	1	4	>128	nd
C. albicans	F5	0.5	0.25	1	>128	nd
C. albicans	Gu5	0.25	1	2	>128	nd
C. albicans	CA1	0.25	0.25	1	2	nd
C. albicans	CA3	0.125	0.25	1	2	nd
C. albicans	CA4	0.125	0.25	0.5	8	nd
C. albicans	11e	1	0.5	2	1	nd
C. albicans	11f	1	0.25	2	1	nd
C. albicans	24g	0.25	0.25	1	1	nd
C. albicans	28A	0.125	0.25	2	>128	nd
C. albicans	28D	0.125	0.25	1	>128	nd
C. albicans	28I	0.125	0.5	1	>128	nd
C. krusei	CK1	0.125	0.5	4	2	nd
C. tropicalis	CT2	0.5	0.5	2	2	nd
C. parapsilosis	CP001	0.125	0.5	2	1	nd
C. neoformans	108	0.25	0.031	0.031	2	nd
A. fumigatus	1161	0.5	0.125	0.5	>64	0.25

a nd, non-detected.

### 19ak exhibits low cytotoxicity with a high selectivity index.

The potential cytotoxicity of these derivatives was evaluated in the human renal epithelial cell line HK2, human bronchial epithelial cell line 16HBE, and human hepatocellular carcinoma cell line HepG2. 19ak and 19k had minimal cytotoxicity in HK2 cells, with IC_50_ values of 26.79 μg/mL and 63.88 μg/mL and high selectivity indexes of 214.32 and 63.88, respectively. By comparison, 19t had much higher cytotoxicity, with an IC_50_ of 3.459 μg/mL. Similar trends were observed for 16HBE and HepG2 cells (Table 3). In general, 19ak and 19k exhibited high selectivity indexes, providing support for their safe application *in vivo*. Given its potent antifungal activity and high safety index, 19ak was chosen for further investigation.

**TABLE 3:** Cytotoxicity of 19ak and its two derivatives against HK2, 16HBE, and HepG2 cells

Compound	IC_50_ (μg/mL)			Selectivity index^*a*^		
	HK2	16HBE	HepG2	HK2	16HBE	HepG2
19ak	26.79	6.972	10.3	214.32	55.78	82.4
19k	63.88	36.43	28	63.88	36.43	28
19t	3.459	1.429	1.389	6.918	2.858	2.778

a The selectivity index is a ratio of IC_50_ against mammalian cell line to MIC_80_ against C. albicans SC5314.

### 19ak chelates fungal intracellular iron and zinc to exert antifungal activity.

**FIG 1:**
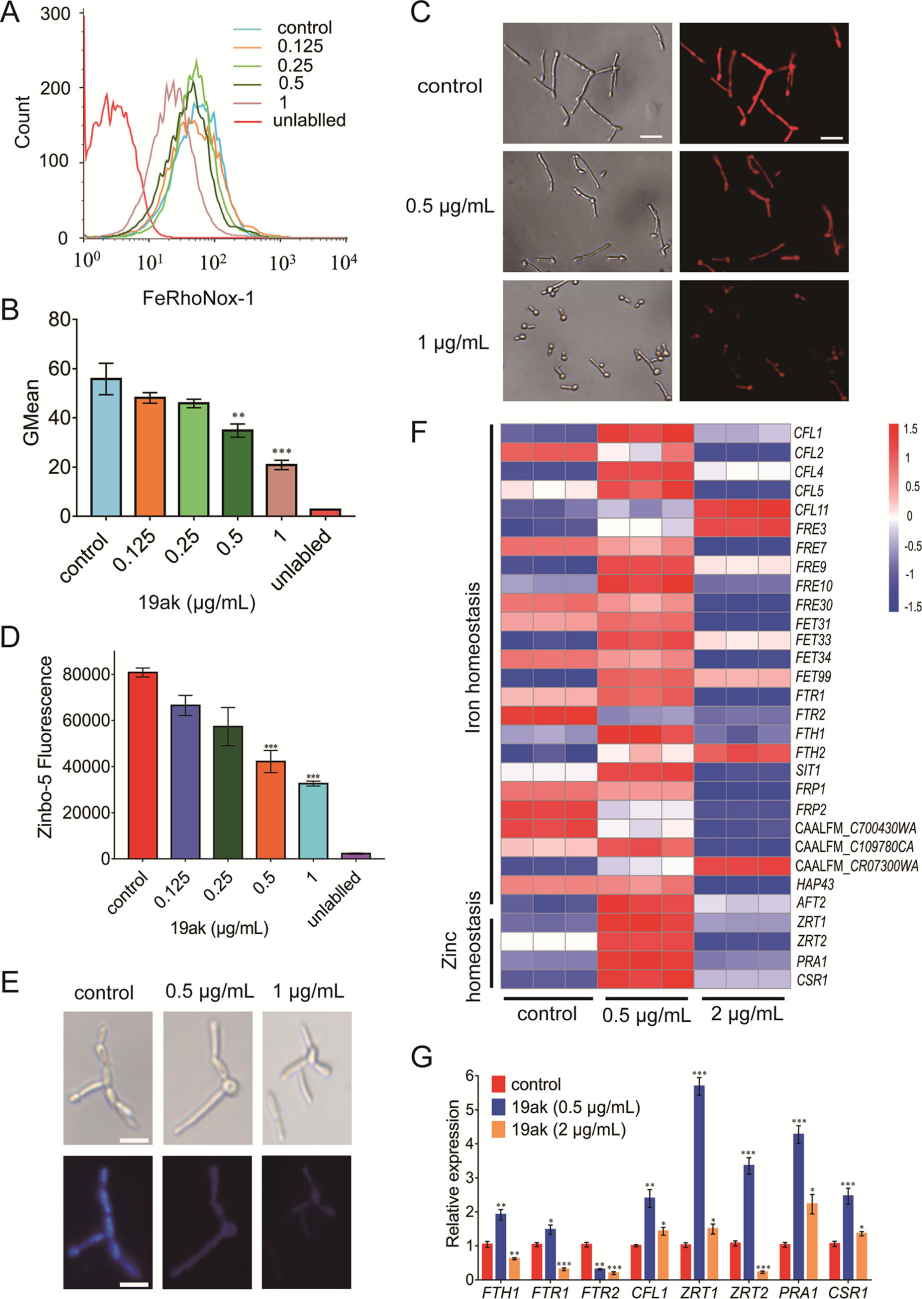
Effects of 19ak on fungal intracellular levels of iron and zinc. (A to C) C. albicans SC5314 at an initial concentration of 1 × 10^6^ cells/mL was treated with 19ak (0.125, 0.25, 0.5, 1 μg/mL) in RPMI 1640 medium at 30°C for 6 h. The cells were then immobilized and stained with 5 μM FeRhoNox-1 for 30 min. The fluorescence intensity of the stained cells was measured by flow cytometry (A) to determine intracellular ferrous iron content, and GMean values were calculated (B), FeRhoNox-1-stained C. albicans cells were further observed by fluorescence microscopy (C) (scale bars: 20 μm). (D, E) The decrease in the fluorescent signal of Zinbo-5 indicates that 19ak chelates intracellular zinc ions. C. albicans SC5314 cells (OD_600_ = 2.0) were treated with various concentrations of 19ak for 6 h in 96-well plates, followed by staining with 10 μM Zinbo-5 for intracellular zinc measurement. The fluorescence intensity was measured by a plate reader (excitation at 355 nm, emission at 485 nm) and Zinbo-5-stained cells were visualized by confocal laser scanning microscopy. (F) Heatmap of the transcriptional expression of genes associated with maintenance of iron and zinc homeostasis under 19ak treatment (0.5 μg/mL or 2 μg/mL). Three replicates were performed. (G) qPCR analysis of the expression of genes associated with iron and zinc homeostasis after 19ak treatment. C. albicans SC5314 was cultured in RPMI 1640 medium and treated with 0.5 μg/mL or 2 μg/mL 19ak for 6 h at 30°C for qPCR analysis. The bars represent means ± SD. *, *P < *0.05; *, *P < *0.01; ***, *P < *0.001.

Thiosemicarbazone derivatives have been reported to chelate iron and zinc (34). To assess the ability of 19ak to chelate iron and zinc in fungal cells, we monitored the intracellular zinc and iron levels of C. albicans cells exposed to 19ak by using fluorescent probes FeRhoNox-1 and Zinbo-5, which specifically bind free ferrous ions and zinc, respectively (21, 35). At a dose of 0.5 μg/mL, 4-fold higher than its MIC value, an obvious drop in intracellular labile ferrous ions was observed in C. albicans SC5314 cells compared with untreated cells (Fig. 1A to C), suggesting potential binding of 19ak to ferric ions or protein-bound iron. However, we were unable to evaluate the ability of 19ak to bind ferric ions and protein-bound iron due to a lack of commercial probes. Zinbo-5 fluorescence staining showed that labile zinc ions decreased upon 19ak treatment at a dose of 0.125 μg/mL. At 1 μg/mL of 19ak, intracellular zinc ions were reduced by approximately 59.6% (Fig. 1D and E).

To further confirm that 19ak exerts its antifungal effects by targeting iron and zinc ion homeostasis, we investigated the effect of extra addition of metal ions on 19ak activity. In the presence of ZnSO_4_ or FeSO_4_, the MIC value of 19ak obviously increased. In the presence of 50 μM ZnSO_4_, the MIC of 19ak against C. albicans SC5314 increased significantly from 0.125 μg/mL to 4 μg/mL (Table 4). In the presence of 50 μM FeSO_4_, the MIC of 19ak against C. albicans SC5314 also increased by 8-fold. The rescue effect of FeCl_3_ was not as obvious as that of FeSO_4_, suggesting that 19ak-mediated antifungal activity depends more on binding ferrous ions than ferric ions (Table 4).

**TABLE 4:** The influence of exogenous metal ions on the MIC value of 19ak against C. albicans SC5314

Compound	MIC (μg/mL)
19ak	0.125
19ak + 50 μM Fe^3+^	0.25
19ak + 100 μM Fe^3+^	0.25
19ak + 200 μM Fe^3+^	0.5
19ak + 50 μM Fe^2+^	1
19ak + 100 μM Fe^2+^	1
19ak + 200 μM Fe^2+^	1
19ak + 50 μM Zn^2+^	4
19ak + 100 μM Zn^2+^	4
19ak + 200 μM Zn^2+^	4

### Transcriptional analysis reveals that 19ak mainly affects oxidoreductase activity, oxidative phosphorylation, and ribosome biogenesis.

To determine the downstream effects of iron and zinc chelation by 19ak on fungal cellular functions at the transcript level, we performed RNA-seq of C. albicans SC5314. In cells treated with 0.5 μg/mL 19ak, 2,632 genes were differentially expressed compared with the control group, of which 1,347 were upregulated and 1,285 were downregulated. Treatment with a high dose (2 μg/mL) of 19ak resulted in the differential expression of 3,335 genes, of which 1,700 were upregulated and 1,635 were downregulated (|log2FoldChange| > 1, *P*-value < 0.05). The differentially expressed genes (DEGs) in common between the high- and low-dose groups were analyzed by using a Venn diagram (Fig. S1). We first focused on genes associated with iron and zinc uptake. Genes involved in iron and zinc acquisition were strongly induced at the low dose, probably as part of regulatory responses, but were downregulated at the high dose (Fig. 1F). These results were further confirmed by qPCR (Fig. 1G).

**FIG 2:**
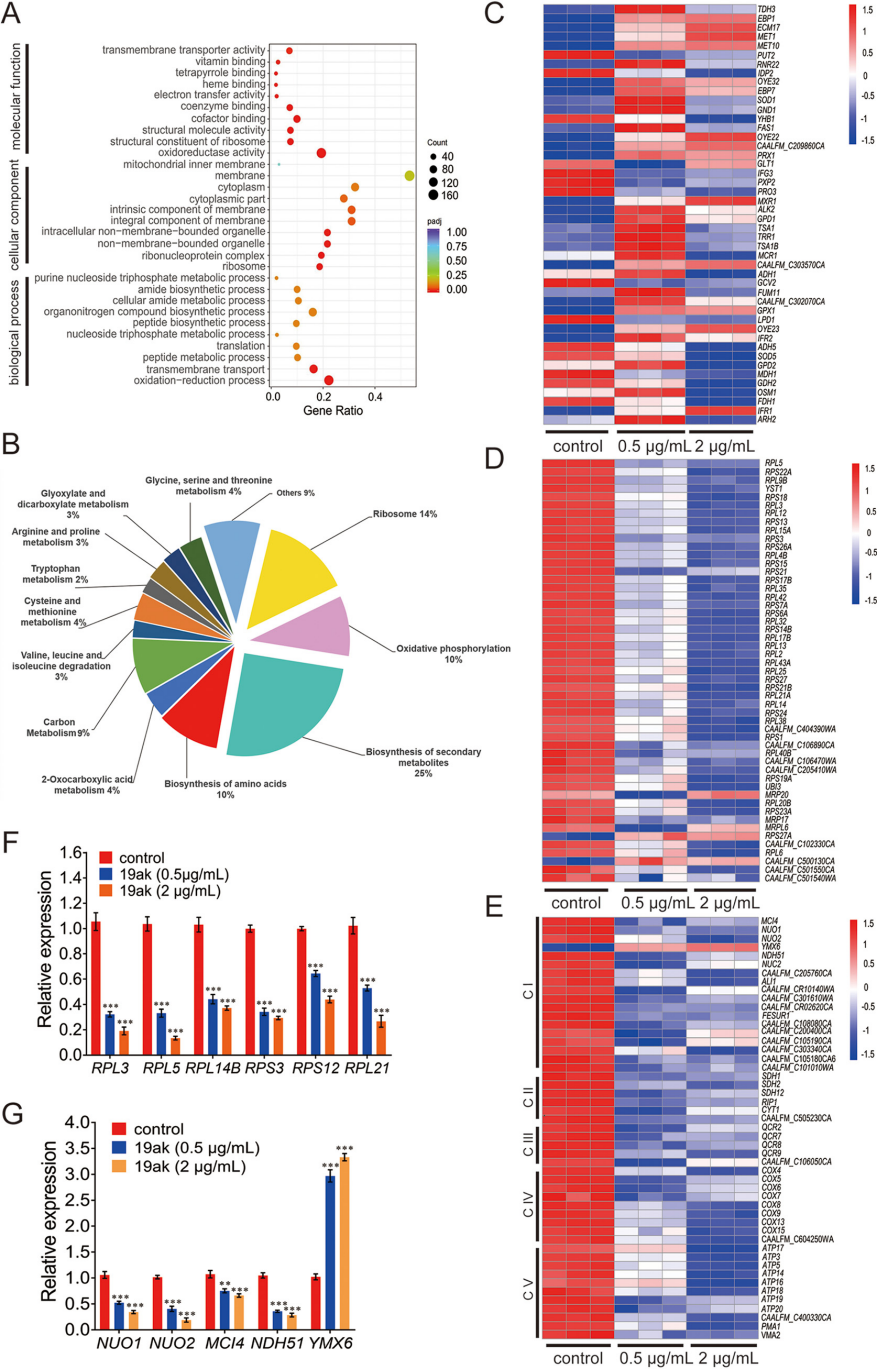
Transcriptional profiling of C. albicans in response to 19ak treatment. (A) Scatterplot of GO enrichment analysis. The size of the scattered points represents the number of DEGs, and the color from red to purple represents the significance of the enrichment. (B) DEGs were annotated using KEGG; functional categories are indicated as percentages of the total number. (C to E) Heatmap of the expression of genes associated with oxidation-reduction process (C), ribosome biogenesis (D), and oxidative phosphorylation (E). The expression levels of genes as log_2_ (FPKM) values are indicated by the color, with red and blue corresponding to high and low expression, respectively. The heatmap includes data from three replicates. Oxidative phosphorylation related genes included those belonging to mitochondrial respiratory chain complex I (CI), complex II (CII), complex III (CIII), complex IV (CIV), and complex V (CV). (F, G) q-PCR analysis of the expression of genes associated with ribosome biogenesis (F) and (G) mitochondrial respiratory complex I after 19ak treatment.

To characterize the cellular pathways affected by C. albicans treatment, GO ontology enrichment analysis of the DEGs was performed. The largest enrichment of DEGs was in the category of oxidation-reduction process for biological processes (GO: 0055114). In terms of molecular function, the categories of oxidoreductase activity, cofactor binding transporter activity, coenzyme binding, and structural constituent of ribosome were notably enriched. At the cellular component level, the ribosome, ribonucleoprotein complex, and non-membrane-bounded organelle categories were significantly enriched (Fig. 2A). We also utilized the Kyoto Encyclopedia of Genes and Genomes (KEGG) pathway database to analyze whether the DEGs belonged to specific signaling pathways. The signaling pathways affected by 19ak mainly included ribosome, oxidative phosphorylation, biosynthesis of secondary metabolites, and amino acid metabolism (Fig. 2B). Collectively, these results suggest that oxidation-reduction process, ribosome components and oxidative phosphorylation are mainly influenced by 19ak. Heatmaps of genes associated with oxidation-reduction process, ribosome components, and oxidative phosphorylation are shown in Fig. 2C to E. Several genes associated with ribosome biogenesis and oxidative phosphorylation were chosen for qPCR analysis (Fig. 2F and G), and the results were consistent with the RNA-seq data.

### 19ak inhibits C. albicans respiratory activity by binding iron.

**FIG 3:**
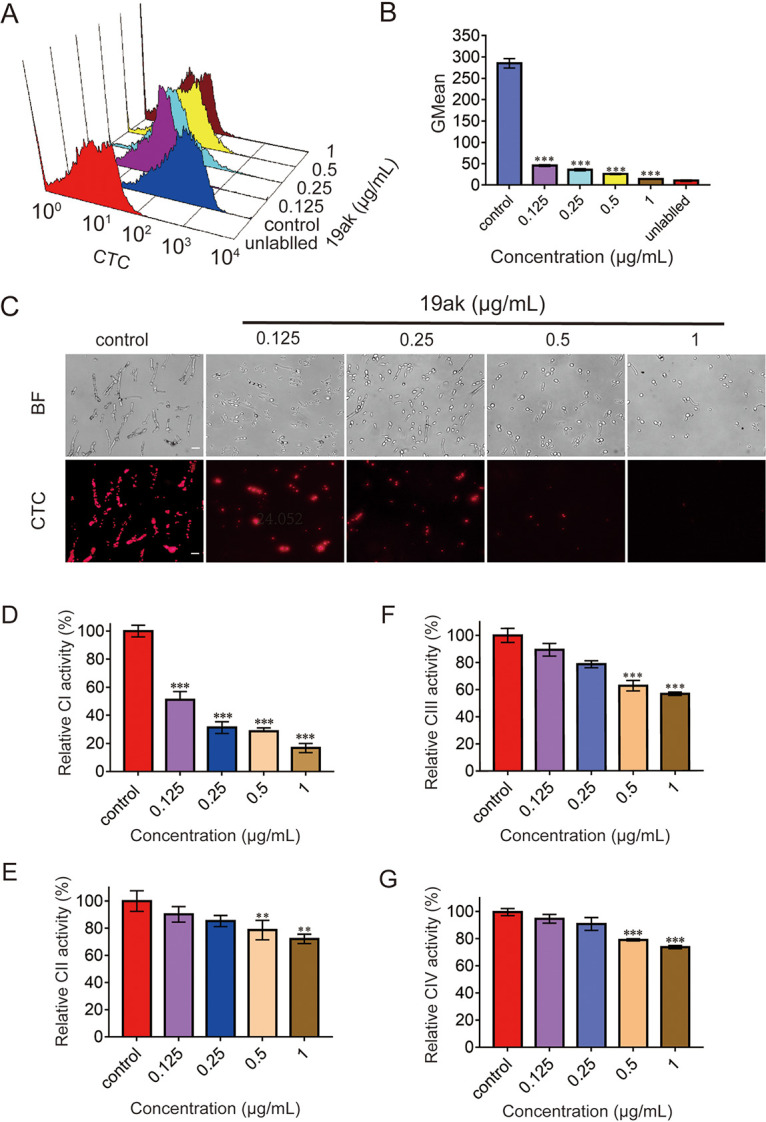
Effect of 19ak on the respiratory activity of C. albicans. (A-C) C. albicans SC5314 (1 × 10^6^ cells/mL in RPMI 1640 medium) was treated with 19ak (0.125, 0.25, 0.5, 1 μg/mL) for 6 h and stained with 1 mM CTC to measure respiratory activity. The fluorescence intensity of the stained cells was measured by flow cytometry (A), and GMean values were calculated (B). Meanwhile, CTC-stained cells were visualized by confocal laser scanning microscopy (C). The scale bar indicates 20 μm. BF, bright field. (D to G) Mitochondria were isolated from C. albicans SC5314 and treated with various concentrations of 19ak (0.125, 0.25, 0.5, 1 μg/mL) for 6 h. The effects of 19ak on the activities of mitochondrial respiratory chain complexes I, II, III, and IV were evaluated according to the protocols of the Micro Mitochondrial Respiratory Chain Complex Activity assay kit.

Due to the close link between oxidoreductase activity and mitochondrial respiration, and the downregulated expression of oxidative phosphorylation-related genes under the treatment of 19ak, we evaluated the effects of 19ak treatment on mitochondrial respiratory activity using the probe 5-cyano-2,3-ditolyl-tetrazolium chloride (CTC). Respiring microbes take up CTC and reduce it to the insoluble fluorescent product formazan, which accumulates in cells and emits red fluorescence upon excitation (36). Both flow cytometry analysis and fluorescence microscopy revealed strong red fluorescence of CTC-stained C. albicans SC5314 cells in the absence of drug treatment, and this fluorescence intensity was dose-dependently reduced in the presence of 19ak, indicating that the respiratory activity of C. albicans was retarded by 19ak (Fig. 3A to C). However, we failed to observe reduced mitochondrial respiration upon treatment with the zinc ion-chelating agent N,N,N′,N′-tetrakis (2-pyridylmethyl) ethylenediamine (TPEN), suggesting that the inhibition of mitochondrial respiration by 19ak was mediated by iron chelation rather than zinc chelation (Fig. S2).

To assess the possible direct effect of 19ak on NADH dehydrogenase activity, *in vitro* assays were performed using purified yeast mitochondria. 19ak significantly inhibited CI in a dose-dependent manner in C. albicans and slightly inhibited the activity of mitochondrial respiratory chain complex III (Fig. 3D to G). When extra metal ions were added to the reaction system, the reduction of NADH dehydrogenase activity induced by 19ak was partially restored (Fig. S3). The activity of CI was significantly inhibited by 19ak at a dose of 0.125 μg/mL, which is lower than the dose at which intracellular free ferrous ions were chelated (Fig. 1B and Fig. 3D). Thiosemicarbazones not only can remove labile iron from cells but can also interact with protein-bound iron to influence diverse biological pathways (37). These results suggest that 19ak directly inhibits CI, possibly by disrupting the normal function of Fe-S clusters. The transcriptional analysis indicated that 48 genes involved in mitochondrial respiratory chain complexes were downregulated after 19ak exposure (Fig. 2E). The gene *YMX6*, which encodes an alternative NADH dehydrogenase in C. albicans, was upregulated by 19ak exposure, possibly as a response to inhibition of CI (Fig. 2G).

### 19ak disrupts mitochondrial function in C. albicans.

**FIG 4:**
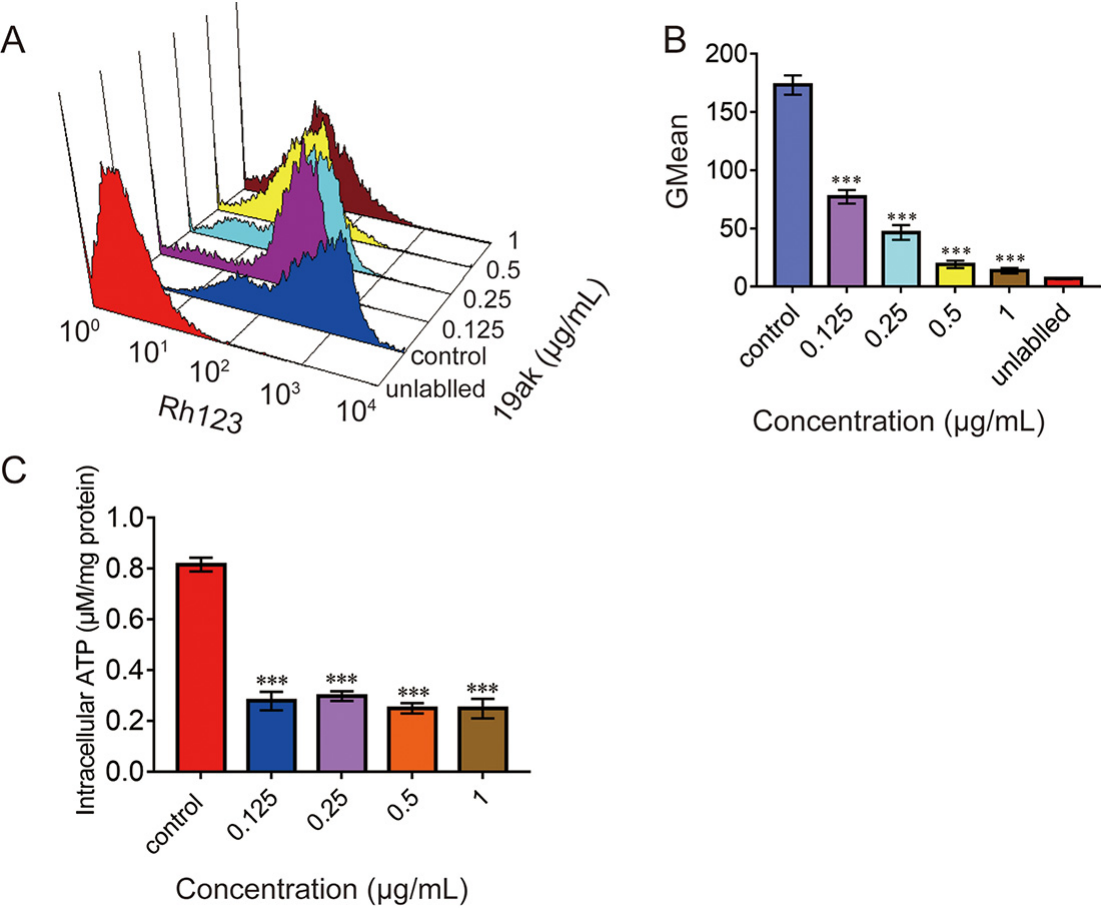
Effect of 19ak on fungal mitochondrial function. (A, B) The effect of 19ak on C. albicans mt*Δψ*. C. albicans SC5314 cells (1 × 10^6^ cells/mL in RPMI 1640 medium) were treated with various doses of 19ak (0.125, 0.25, 0.5, 1 μg/mL) at 30°C for 6 h. The cells were then stained with 20 μM Rh123 for 30 min in the dark and detected by flow cytometry. GMean values were calculated. (C) The effect of 19ak on ATP generation. C. albicans SC5314 cells were treated with 19ak at the indicated concentrations (0.125, 0.25, 0.5, 1 μg/mL) and disrupted in lysis buffer. Intracellular ATP was detected using an ATP assay kit. The bars represent the means ± SD of three independent experiments. *, *P < *0.05;*, *P < *0.01; ***, *P < *0.001.

Mitochondrial membrane potential (mt*Δψ*) is an indicator of the energy status of mitochondria and can be used to evaluate the activity of proton pumps and electrogenic transport systems (38). ATP synthesis is another indicator of mitochondrial function. To further probe the effect of 19ak on mitochondrial function, we first utilized Rh123 to measure mt*Δψ* in C. albicans. 19ak reduced the fluorescence intensity of Rh123 (Fig. 4A and B), indicating a decrease in mt*Δψ*. Moreover, 19ak significantly inhibited intracellular ATP production; 0.125 μg/mL 19ak decreased intracellular ATP levels by approximately 65.8% compared with control cells (Fig. 4C).

### The antifungal activity of 19ak is also mediated by reductive stress.

**FIG 5:**
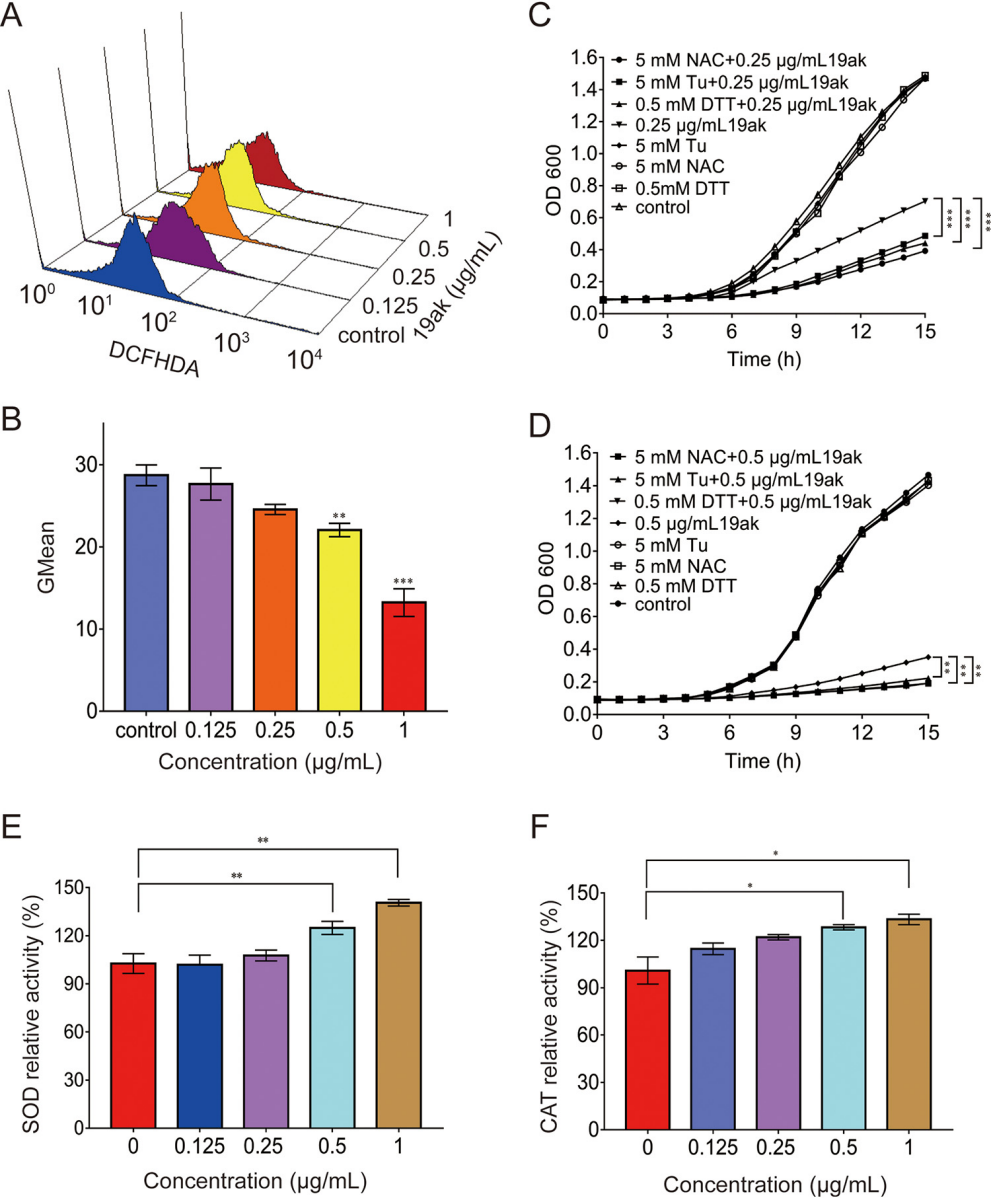
19ak elicits reductive stress in C. albicans. (A, B) ROS production in C. albicans under 19ak treatment. C. albicans SC5314 cells (1 × 10^6^ cells/mL in RPMI 1640 medium) were treated with 19ak for 6 h and stained with 40 μg/mL 2,7-dichlorofluorescein diacetate (DCFHDA, Sigma-Aldrich, USA) for ROS measurement. The fluorescence intensity of DCFHDA-stained cells was evaluated by flow cytometry, and GMean values were calculated. (C, D) C. albicans SC5314 cells were adjusted to 1 × 10^5^ cells/mL in SC medium and treated with 0.25 μg/mL (C) or 0.5 μg/mL (D) 19ak. NAC, Tu, or DTT was added as an antioxidant agent at the indicated concentrations and incubated at 30°C for 15 h. Fungal cell growth was monitored by measuring the optical density at 600 nm. The OD_600_ values at the final detection point were used to analyze significant differences. (E, F) The effect of 19ak on the activity of SOD or CAT. C. albicans SC5314 cells were incubated with various doses of 19ak at 30°C for 6 h. The cells were then lysed, and SOD or CAT activity was measured by using a SOD assay kit or CAT assay kit, respectively. *, *P < *0.05; *, *P < *0.01; ***, *P < *0.001.

ROS are a by-product of mitochondrial respiration. Given the influence of 19ak on respiration, we utilized the fluorescent probe DCFH-DA to monitor ROS formation in C. albicans (39). Flow cytometry analysis showed that 19ak treatment decreased ROS in C. albicans cells compared with the control, indicating reductive stress (Fig. 5A and B). To elucidate the role of 19ak-induced reductive stress, we investigated the effects of H_2_O_2_, N-acetyl-L-cysteine (NAC), and thiourea (Tu) on the antifungal activity of 19ak. As shown in Table S3, the MIC value of 19ak increased by 2- to 4-fold after 24 h in the presence of different concentrations of H_2_O_2_. By contrast, the antioxidants NAC and Tu greatly enhanced the antifungal activity of 19ak based on cell growth monitoring (Fig. 5C and D).

The excessive accumulation of reducing equivalents or increased activity of antioxidant enzymes is considered reducing pressure and can overwhelm endogenous oxidoreductase activity (40). The RNA-seq data indicated upregulation of the transcription of several genes associated with oxidoreductase activity (Fig. 2C), implying that in addition to reducing ROS generation, 19ak-induced antioxidant enzymes may contribute to reductive stress. We therefore assessed the activities of CAT and SOD. Treatment with 19ak at 0.5 μg/mL or above increased CAT and SOD activities, suggesting that the induction of antioxidant enzymes is an important source of reductive stress caused by 19ak (Fig. 5E and F). Similar to oxidative stress, reductive stress impairs cellular functions such as endoplasmic reticulum (ER) stress (41). As expected, 19ak-treated C. albicans cells exhibited higher hypersusceptibility to the ER stress inducer dithiothreitol (DTT) (Fig. 5C and D).

### 19ak inhibits C. albicans ribosome biogenesis mainly through disruption of intracellular zinc homeostasis.

Fe^2+^ is a ribosomal cofactor and is required for normal ribosome function (42). To distinguish the effects of iron or zinc chelation by 19ak on ribosome biogenesis, we assessed the transcriptional expression levels of ribosome-related genes in C. albicans treated with the iron-specific chelators CPX and deferoxamine (DFO). CPX had no obvious effect on the expression of the evaluated genes (Fig. S4A). DFO only had minor effects on reducing the expressions of several genes associated with ribosome biogenesis compared with 19ak (Fig. S4B). These results suggest that disruption of intracellular iron homeostasis has a minor effect on ribosome biogenesis.

**FIG 6:**
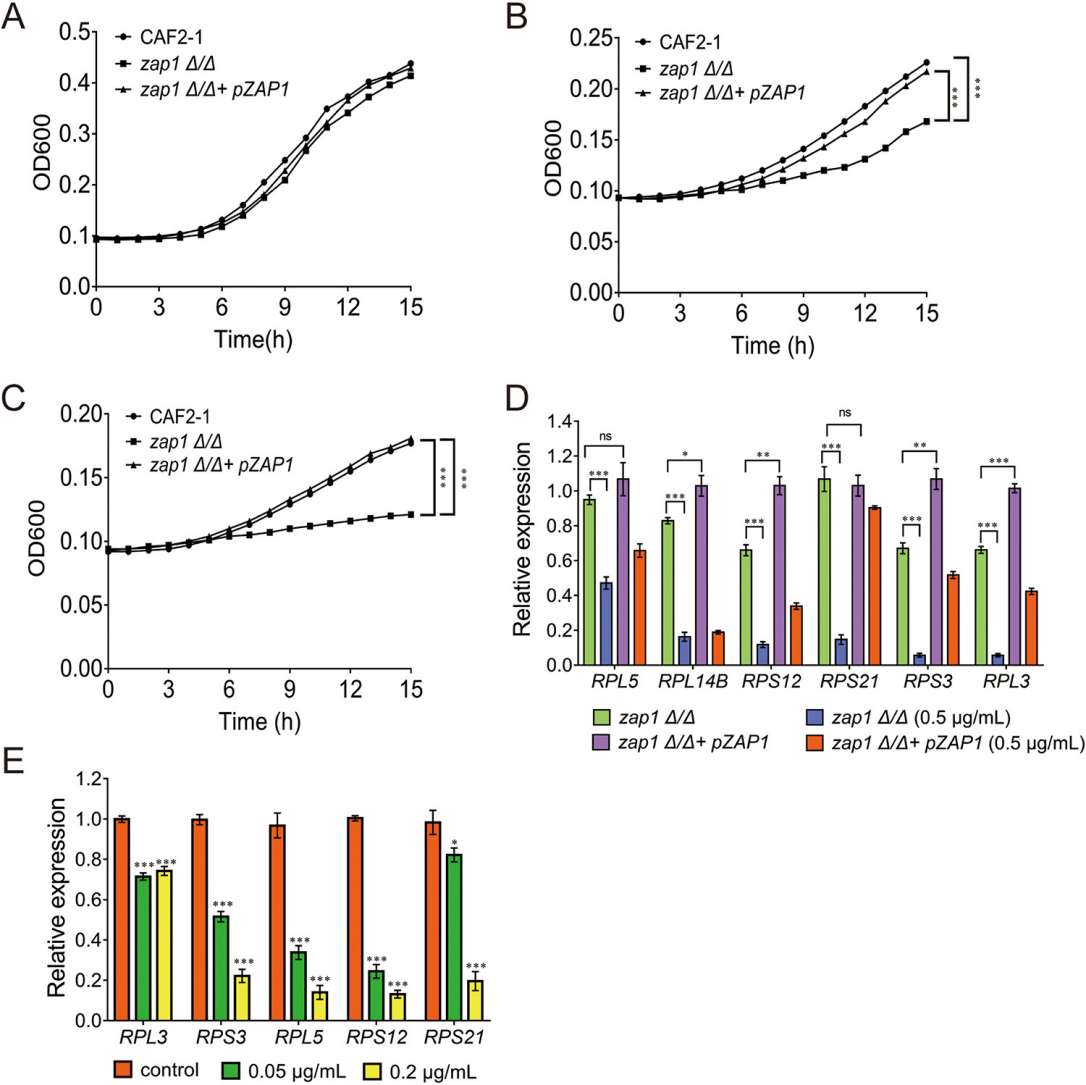
Effect of 19ak on ribosome biogenesis in C. albicans. (A to C) The wild strain CAF2-1, the mutant strain *zap1Δ/Δ* and its complemented strain *zap1Δ/Δ*+ p*ZAP1* were adjusted to 1 × 10^5^ cells/mL in RPMI 1640 medium and treated with 0.25 or 0.5 μg/mL 19ak for 15 h at 30°C. Cell growth was monitored by detecting the optical density at 600 nm in a microplate reader. (A) Cells that were not treated with 19ak as a control. (B) Cells treated with 0.25 μg/mL 19ak. (C) Cells treated with 0.5 μg/mL 19ak. Each point represents the mean of three replicates. The OD_600_ values at the final detection point were used to analyze significant differences. (D) C. albicans *zap1Δ/Δ* and its complemented strain *zap1Δ/Δ* + p*ZAP1* were treated with 0.5 μg/mL 19ak for 6 h at 30°C. The transcription levels of several genes associated with ribosomal biosynthesis were determined by qPCR. The bars represent means ± SD from three independent experiments. *, *P < *0.05; **, *P < *0.01; ***, *P < *0.001. ns, nonsignificance. (E) qPCR analysis of the expression of genes associated with iron and zinc homeostasis after TPEN treatment. C. albicans SC5314 was cultured in RPMI 1640 medium and treated with 0.05 μg/mL or 0.2 μg/mL TPEN for 6 h at 30°C for qPCR analysis.

Zinc is a cofactor for many enzymes, and maintaining intracellular zinc homeostasis is therefore critical for normal cell growth. The ribosome is considered a zinc store, and in some bacteria, the transcription of ribosomal proteins is regulated by the zinc-specific regulator Zur (43–45). However, little is known about the relationship between zinc homeostasis and ribosome biogenesis in fungal cells. In yeast, the transcription factor Zap1 regulates adaptive responses to zinc deficiency and is considered a central player in zinc homeostasis (46). Therefore, to investigate whether 19ak-mediated ribosome biogenesis inhibition is caused by zinc deprivation, we tested the susceptibility of a *zap1* mutant of C. albicans to 19ak by measuring cell growth. In the absence of 19ak, the growth profile of the *zap1* mutant was similar to that of the wild-type strain CAF2-1 and complemented strain. However, when treated with 19ak, the *zap1* mutant exhibited more severe growth defects than the complemented strain (Fig. 6A to C), suggesting that disruption of intracellular zinc regulation aggravates the growth defect under zinc deficiency caused by 19ak. In addition, when exogenous 100 μM Zn^2+^ was added, 19ak lost the activities against all three strains (Fig. S5). To test whether ribosome biogenesis is affected by zinc deficiency, we evaluated the transcriptional expression of ribosome-associated genes in the *zap1* mutant in the presence or absence of 19ak compared with the complemented strain. qPCR showed that several genes associated with ribosome biogenesis were indeed downregulated in the *zap1* mutant compared with the complemented strain, and this downregulation was even more evident in *zap1* mutant cells treated with 19ak (Fig. 6D). Moreover, we observed that the expression of genes associated with ribosome biogenesis was also downregulated by another known zinc chelator TPEN (Fig. 6E). These observations suggest that ribosome biogenesis is regulated by zinc homeostasis.

### 19ak exhibits selectivity in chelating metal ions between fungi and mammalian cells.

**FIG 7:**
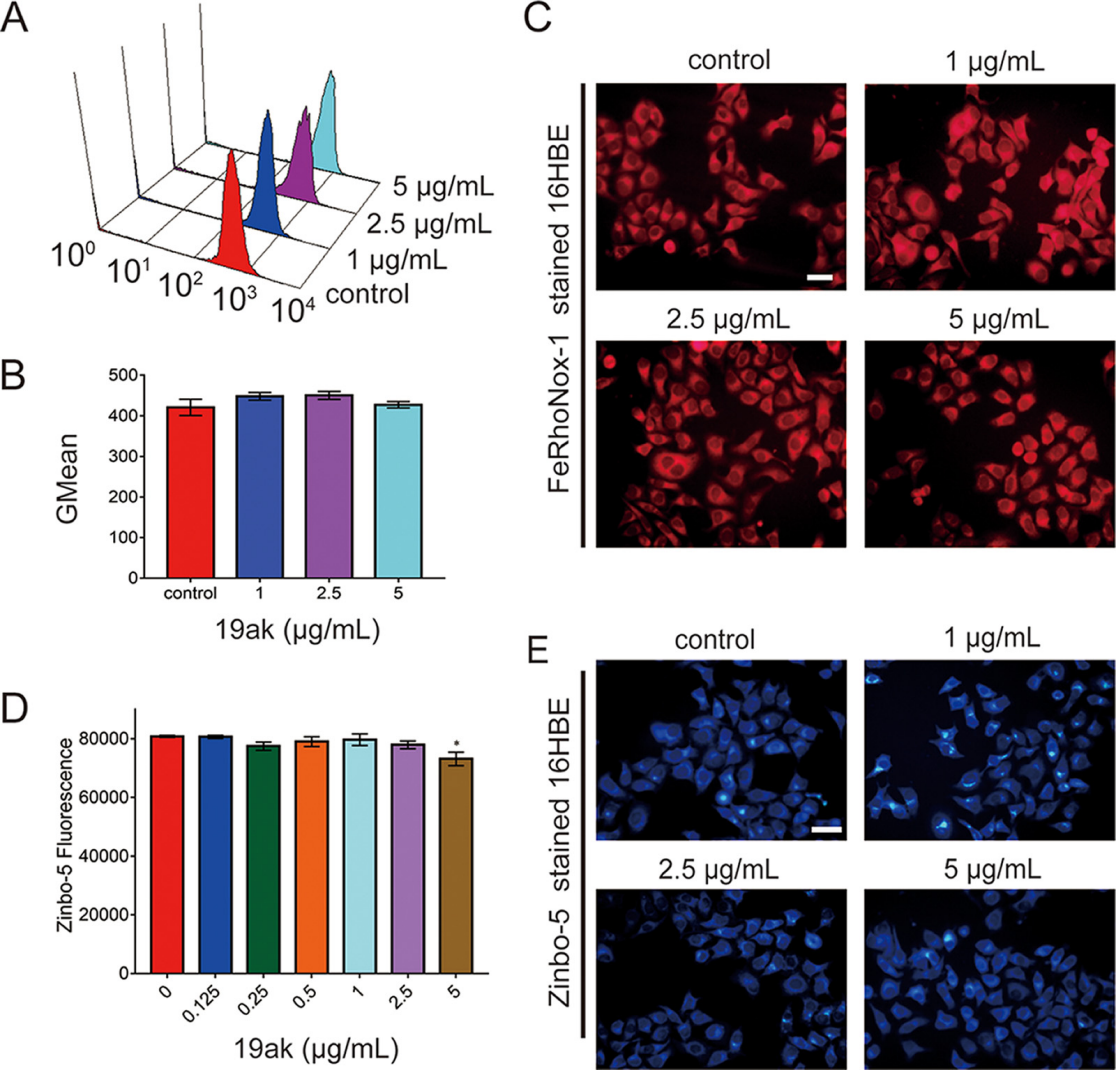
The effect of 19ak on metal chelation in mammalian 16HBE cells. (A to C) Mammalian 16HBE cells were treated with 19ak for 12 h. The cells were then stained with FeRhonox-1 for measurement of fluorescence intensity by flow cytometry (A). The GMean value of fluorescence intensity was calculated (B). FeRhoNox-1-stained cells were further observed by fluorescence microscopy (scale bars: 20 μm) (C). (D, E) Mammalian 16HBE cells were treated with 19ak for 12 h. The cells were then stained with 10 μM Zinbo-5, the fluorescence intensity was measured by a plate reader (D) or observed by fluorescence microscopy (E).

To determine the potential factors for low cytotoxicity of 19ak, we measured the intracellular contents of ferrous and zinc in mammalian cells under the treatment of 19ak. Compared with the significant decrease in ferrous ion and zinc ion in C. albicans, 19ak almost had no effects on ferrous and zinc contents in mammalian cells including 16HBE and human liver cancer cells HepG2 at concentrations of 5 μg/mL, which is 40-fold higher than the MIC value against C. albicans SC5314. (Fig. 7, Fig. S6). We further evaluated the differential effects of 19ak on mitochondrial dehydrogenase activity between fungal cells and mammalian cells using a Cell Counting Kit-8 (CCK-8) reduction assay. The reagent WST-8 contained in CCK-8 can be converted to a water-soluble orange formazan product by dehydrogenase in the cell. The orange color became faint when the concentration of 19ak increases from 0.125 μg/mL to 5 μg/mL in C. albicans and the absorbance at 450 nm decreased accordingly in a dose-dependent manner. However, 19ak even at 5 μg/mL exhibited minimal inhibitory effect on the activity of mammalian cells mitochondrial dehydrogenase after either 12 h or 48 h of treatment (Fig. S7).

### 19ak exhibits low potential to induce drug resistance.

The widespread use of azole drugs increases the likelihood of development of resistance of C. albicans to these agents. To assess the capacity of 19ak to induce the development of resistance in C. albicans, C. albicans SC5314 cells were repeatedly exposed to 19ak or FLC in RPMI 1640 medium over a 40-day period. Repeated treatment with 19ak did not alter the MIC of 19ak, whereas repeated treatment with FLC resulted in a significant increase in the MIC value of FLC (Fig. S8). These results suggest that 19ak has minimal propensity to induce resistance in C. albicans probably due to a lack of energy to support resistance mechanisms in treated cells.

### 19ak exhibits antifungal therapeutic efficacy *in vivo*.

**FIG 8:**
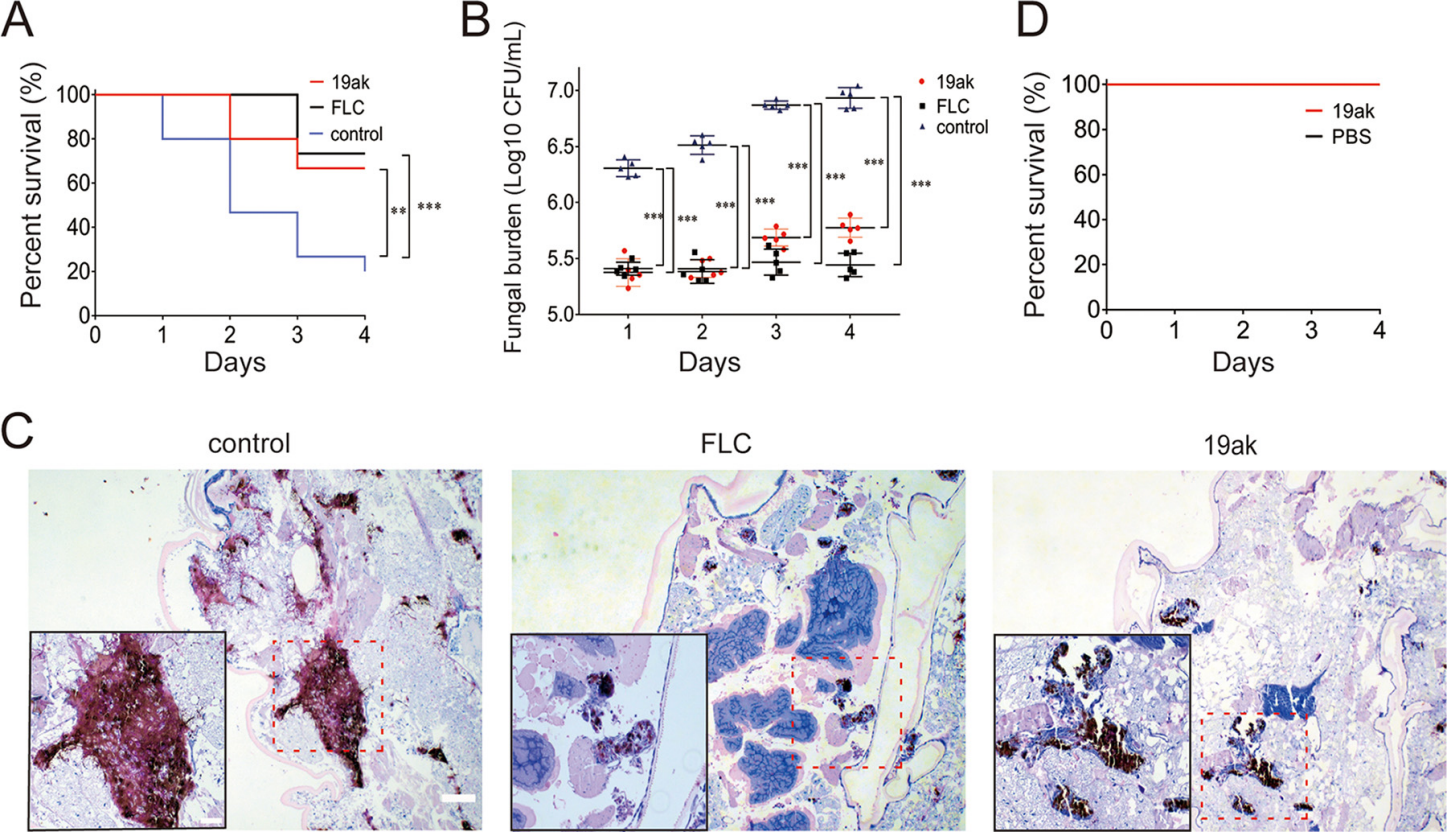
19ak exhibits therapeutic antifungal efficacy in a G. mellonella infection model. Larvae were infected with 2 × 10^5^ CFU/larva of C. albicans SC5314 and treated with PBS (control), FLC (2 μg/larva), or 19ak (0.4 μg/larva) after 2 h of infection. (A) Survival curves of G. mellonella infected with C. albicans under different drug treatments (*n* = 15 per group). (B) The fungal burden of G. mellonella infected with C. albicans under different drug treatments was monitored every day after infection. *, *P < *0.05; **, *P < *0.01; ***, *P < *0.001. (C) Histopathology of G. mellonella infected with C. albicans under different drug treatments. The bars indicate 200 μm. The insets are enlarged images of the areas enclosed in rectangles (marked by dotted lines). (D) The potential toxicity of 19ak. G. mellonella were treated with 10 μg of 19ak per larva. After 4 days, all survived and no notable changes in behavior were observed compared with untreated larvae.

The lepidopteran G. mellonella is a mini-host for *Candida species* (47). Models of G. mellonella infection with *Candida*, Cryptococcus, *Trichosporon*, Aspergillus, and *Mucorales* are widely used to assess fungal virulence and antifungal drug efficiency (48). We utilized G. mellonella to test the *in vivo* antifungal efficacy of 19ak. After 4 days of infection, the survival rate was only 20% in the control group but 67% in the group treated with 19ak (0.4 μg per larva), with similar therapeutic efficacy to that of FLC (2 μg per larva) (Fig. 8A). In addition, 19ak and FLC greatly reduced the fungal burden compared with the control group (Fig. 8B). Histopathology studies revealed that larvae treated with 19ak or FLC had fewer infected areas than untreated larvae, and the number of black nodules was significantly lower in 19ak- or FLC-treated larvae (Fig. 8C). To further assess the potential toxicity of 19ak, G. mellonella was treated with a high dose of 10 μg of 19ak per larva. After 4 days of treatment, all treated larvae were still alive and had no notable changes in behavior compared with untreated larvae, supporting the safety of 19ak for treating fungal infections, at least in the G. mellonella model (Fig. 8D).

## DISCUSSION

Thiosemicarbazones are an important class of compounds with antifungal activities, and numerous compounds or their metal complexes have been synthesized. Thiosemicarbazones primarily inhibit fungal growth, i.e., fungistatic activity, but the activity of an individual compound often varies widely among fungal species (49). For example, a lapachol thiosemicarbazone derivative exhibited potent antifungal activity against isolates of Paracoccidioides brasiliensis but was ineffective against *Candida* species at the tested concentrations (50). This variation in sensitivity is consistent with our findings that some thiosemicarbazone derivatives with stronger anti-*Candida* activity had weaker activity against C. neoformans. The modes of action of thiosemicarbazones generally include iron chelation, inhibition of ergosterol synthesis, or increased ROS generation (51, 52). The effects of thiosemicarbazones on ergosterol synthesis likely reflect their iron-chelating abilities, as iron homeostasis is closely associated with ergosterol synthesis (53). Iron chelators such as CPX interfere with sterol synthesis and reduce ergosterol content in C. albicans (54).

In this study, we screened a library of 89 thiosemicarbazones and determined that the thiosemicarbazone derivative 19ak had the best anti-C. albicans activity, with an MIC value against the wild-type strain 16-fold lower than that of FLC. Derivative 19ak exhibited low cytotoxicity and *in vivo* efficacy for the treatment of candidiasis in a G. mellonella model. These excellent *in vitro* and *in vivo* activities warranted the investigation of the mode of action of 19ak. Cellular assays using fluorescence probes revealed that 19ak exhibited potent iron- and zinc-chelation abilities. Moreover, treatment with a low dose of 19ak upregulated the expression of genes involved in metal uptake, suggesting a feedback effect. Interestingly, the depletion of intracellular iron and zinc by 19ak was minimal in mammalian cells, even at a dose 40 times higher than its MIC value for C. albicans SC5314. These distinct effects of 19ak in mammalian versus fungal cells may explain the good biological safety of 19ak and support its potential application as a selective inhibitor of respiratory activity in C. albicans cells. We previously reported that the iron chelator hinokitiol inhibits mitochondrial respiration and mitochondrial respiratory chain CI and CII activity (55). Consistent with these previous findings, 19ak inhibited mitochondrial respiration and mainly reduced CI activity.

Few studies have investigated the downstream effects of zinc chelation in fungi. In a Gram-positive bacterium Bacillus subtilis, zinc ion deprivation inhibits ribosome translation activity (56). In addition, Sun et al. reported that the thiosemicarbazone derivative NSC319726 exerts fungicidal activity by inhibiting ribosome biogenesis and increasing ROS generation (57). Given these reports, we investigated inhibition of ribosome biogenesis as a possible downstream effect of zinc chelation in C. albicans. We observed that the *zap1* mutant of C. albicans, which has reduced adaptability to zinc deficiency, was sensitive to inhibition by 19ak. Several genes associated with ribosome biogenesis were downregulated in the *zap1* mutant in response to treatment with 19ak or TPEN, another zinc chelator. However, no fungicidal activity was observed until the dose of 19ak was increased to 512 MIC. TPEN also exhibits growth inhibition rather than killing activity against *Candida* species (21), as does the zinc chelator pyrvinium pamoate (58). These observations suggest that inhibition of ribosome biogenesis by zinc chelation only inhibits the growth of *Candida*. However, TPEN has fungicidal activity against Trichophyton rubrum and A. fumigatus conidia (19, 59). In addition, 19ak exhibited minimal ability to develop drug resistance, consistent with the findings for other zinc chelators, such as ZAC307 and ZAC989 (21).

Another notable facet of the mode of action of 19ak is induction of reductive stress. Reductive stress is the result of excessive accumulation of reductants such as NADH, NADPH, and GSH, which disrupts the delicate redox balance in the cell. Imbalance of intracellular redox reactions is a hallmark of a variety of pathophysiological processes (40). For example, a persistent lack of ROS results in reductive stress and causes cardiomyopathy, obesity, or diabetes (60, 61). Treatment with 19ak caused reductive stress by reducing cellular ROS levels due to the inhibition of mitochondrial respiration and increased activities of antioxidant enzymes. Moreover, reduced oxidation of NADH due to inhibition of respiratory chain CI by 19ak may result in accumulation of NADH as another source of reductive stress.

Taken together, our results uncover the mechanisms underlying the inhibition of C. albicans proliferation by the thiosemicarbazone 19ak and support its potential application to treat relevant fungal infections, particularly those resulting from resistant organisms.

## MATERIALS AND METHODS

### Measurement of intracellular free iron levels using FeRhoNox-1.

FeRhoNox-1 is a commercially available fluorescent probe that specifically binds Fe^2+^ and is used to measure intracellular Fe^2 +^ content. C. albicans SC5314 cells cultured overnight were diluted to roughly 1 × 10^6^ cells/mL in RPMI 1640 medium and challenged with various doses of 19ak for 6 h. Next, the cells were fixed with 4% paraformaldehyde for 1 h. After washing three times with PBS, the cells were stained with 5 μM FeRhoNox-1 for 30 min and analyzed by flow cytometry (Becton, Dickinson, CA, USA). Mammalian cells culture is according to the method described in Cytotoxicity testing which was described in Supplemental material in detail. After 12 h of treatment with different concentrations of 19ak, the supernatant was discarded and washed three times with PBS. Cells were collected after staining with FeRhoNox-1 for 30 min in the dark, and the fluorescence intensity of stained cells was analyzed by flow cytometry.

### Measurement of intracellular labile zinc levels using Zinbo-5.

C. albicans SC5314 cells grown overnight were pelleted, washed three times with PBS buffer, and resuspended to obtain an OD600 of 2.0. Next, the cells were incubated with various concentrations of 19ak in 96-well plates at 30°C for 6 h, followed by incubation with 10 μM Zinbo-5 for 30 min. Finally, fluorescence was measured in a microplate reader (excitation at 355 nm, emission at 485 nm). Similar approach was used in detection of intracellular zinc levels of mammalian cells.

### RNA isolation, cDNA library construction, and sequencing.

C. albicans SC5314 cells were inoculated into RPMI 1640 medium at a concentration of 1 × 10^7^ cells/mL and incubated at 30°C for 6 h. The cells were then harvested, and RNA extraction was performed with the Yeast RNA Kit (OMEGA, Guangzhou, China). RNA purity was evaluated using a Nanodrop-2000, and RNA integrity was assessed using the RNA Nano 6000 detection kit. RNA-Seq libraries are prepared using NEB Next Ultra RNA Library Prep Kit (NEB, MA, USA) for Illumina. The cDNA library quality was quantified first by using Qubit 2.0 Fluorometer, detected on Agilent 2100 bioanalyzer. Clustering was performed in the cBot cluster generation system. After cluster generation, the library preparations were sequenced on the Illumina Novaseq platform. Library construction and sequencing were performed by Novogene (Beijing, China). Each sample was analyzed in triplicate.

### RNA-Seq data analysis and differentially expressed genes identification.

The raw data in fastq format were first processed by an internal perl script. In this step, clean data were obtained by applying various processing methods. All subsequent downstream analyses were based on high-quality clean data. The clean reads were individually mapped to the C. albicans SC5314 reference genome using HISAT2 (v2.0.5). The fragments per kilobase of transcript per million mapped reads (FPKM) value was calculated for each gene, and the read counts were mapped to the genes. The DESeq2R package (1.20.0) was used to analyze differences in expression between two groups with biological replication. Genes with an adjusted *P* value < 0.05 and |log2FoldChange| > 1 as detected by DESeq2 were considered DEGs. The RNA sequencing data have been submitted to the Gene Expression Omnibus (GEO) under accession number GSE179537.

### Determination of *in vivo* antifungal effects in the G. mellonella infection model.

The G. mellonella infection model was used to evaluate the *in vivo* activity of 19ak in terms of survival, fungal burden, and histological features. A total of 60 larvae of similar size were selected and randomly allocated to four groups. The last right foreleg of each larva was injected with 10 μL of C. albicans SC5314 suspension (2 × 10^7^ CFU/mL). Two hours after infection, the larvae were injected with 10 μL of drug or vehicle as follows for the four groups: 19ak (0.4 μg per larva), FLC (2 μg per larva), control (PBS), and blank control (no injection with C. albicans but injections of sterile PBS twice). The data for the blank control are not shown because this group survived well during the experiment. Failure to respond to light contact with forceps was considered a sign of larval death. In the survival assay, the number of surviving larvae in each group was calculated at the same time every day for 4 days.

To determine fungal burden, larval infection and treatment were performed as described above with groups of 30 larvae each. Each day, five larvae were randomly selected from each group and homogenized with glass beads in 0.5 mL of sterile PBS. The homogenate was serially diluted with sterile PBS, and 5 μL was spread on a yeast-peptone-dextrose solid plate (62). Experiments were performed at least three times independently.

We also conducted histopathological studies to explore the therapeutic effects of the drugs on G. mellonella fungal infection. First, the groups of larvae were infected as described above. After 48 h, three larvae from each group were randomly selected and fixed in 4% paraformaldehyde for sectioning and Periodic acid-Schiff (PAS) staining. The tissue was observed and photographed under an Olympus microscope with 40 × magnification (IX71, Olympus, Tokyo, Japan). The experiments were performed independently a minimum of three times.

### Evaluation of the toxicity of 19ak.

Larvae of normal size and in good condition were injected with 10 μL of 19ak (1 mg/mL) or PBS. The survival of the larvae was monitored daily for 4 days.

### Data availability.

RNA-Seq data reported in this study have been deposited with the Gene Expression Omnibus under accession number GSE179537. The data that support the findings of this study are available from the corresponding author upon reasonable request.
