# Supplementary material for: Selective Metal Chelation by a Thiosemicarbazone Derivative Interferes with Mitochondrial Respiration and Ribosome Biogenesis in Candida albicans
Source: Microbiol Spectr. 2022 Apr 18;10(3):e01951-21. doi: 10.1128/spectrum.01951-21 (PMC9241695; doi:10.1128/spectrum.01951-21)
Supplement: SUPPLEMENTAL FILE 1 — Supplemental material. Download spectrum.01951-21-s001.pdf, PDF file, 1.8 MB [file spectrum.01951-21-s001.pdf]

Supporting Information

Selective metal chelation by a thiosemicarbazone derivative interferes with mitochondrial respiration and ribosome biogenesis in *Candida albicans*

Ximeng Duan[1], Zhiyu Xie[2], Liying Ma[3], Xueyang Jin[1], Ming Zhang[4], Yuliang Xu[1], Yue Liu[1], Hongxiang Lou[1,*] and Wenqiang Chang [1,*]

TABLE S1. Structures of the thiosemicarbazone derivatives and their MICs against *C. albicans* SC5314.

Compd.	Structure	MIC (μg/mL)
19a	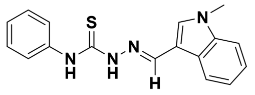	>64
19b	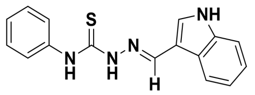	>64
19c	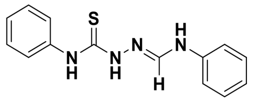	>64
19d	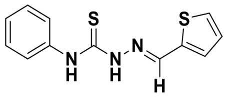	>64
19e	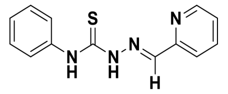	>64
19f	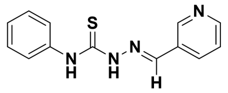	>64
19g	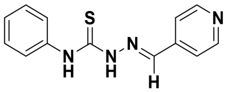	>64
19h	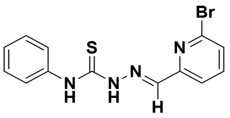	>64
19i	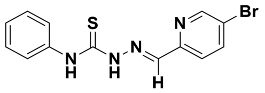	>64
19j	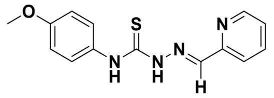	>64
19l	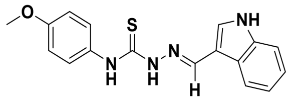	>64
19m	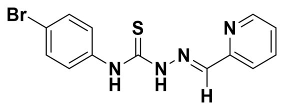	>64
19n	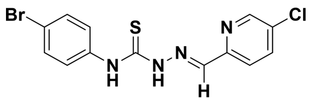	>64

19o	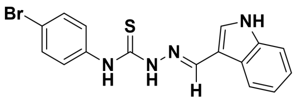	>64
19p	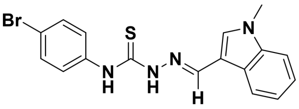	>64
19u	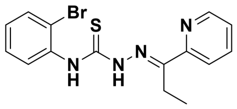	>64
19w	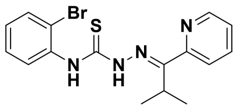	>64
19y	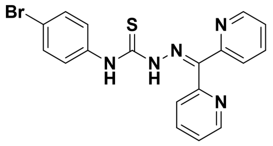	>64
19z	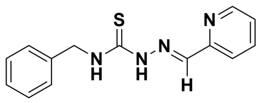	>64
19aa	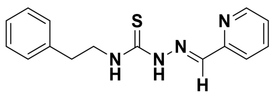	>64
19ad	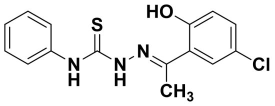	>64
19ae	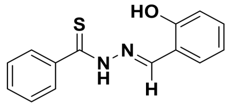	32
19af	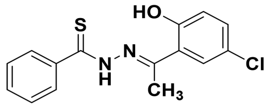	>64
19ah	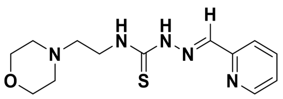	>64
19al	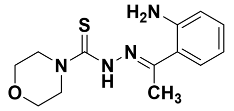	>64
19am	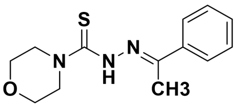	>64
19an	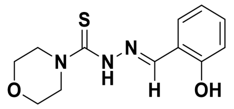	64

19as	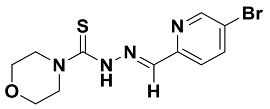	>64
19at	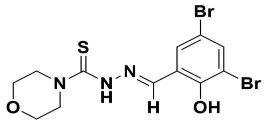	>64
19ap	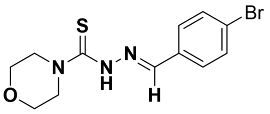	16
19aq	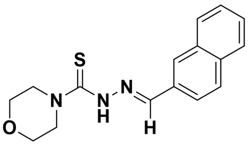	8
19au	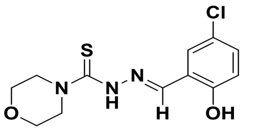	>64
19ar	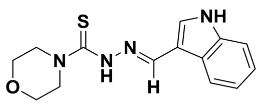	32
19av	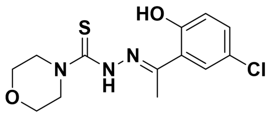	>64
19aw	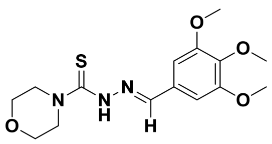	>64
19ay	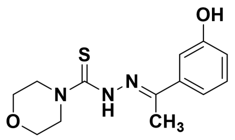	>64
19az	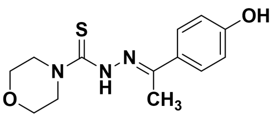	>64
19ai	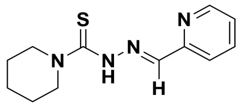	8
19ax	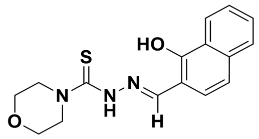	>64
19ba	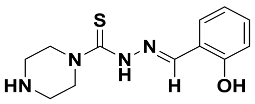	>64

19bb	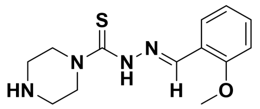	16
19bc	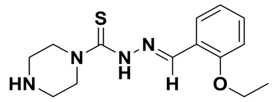	>64
19bd	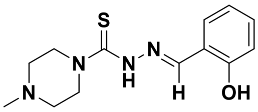	>64
19bf	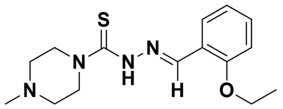	>64
1157	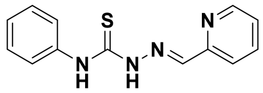	>64
1156	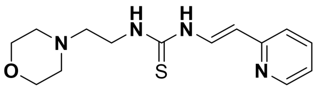	>64
315	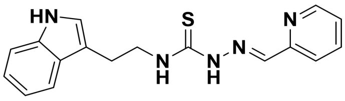	>64
533	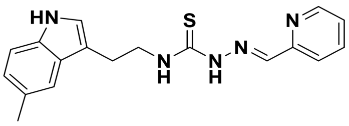	>64
500	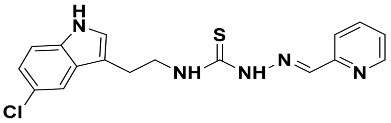	>64
523	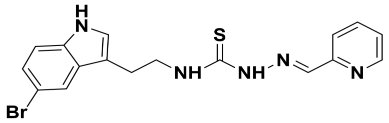	>64
502	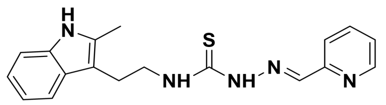	>64
634	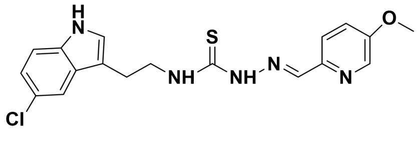	>64
633	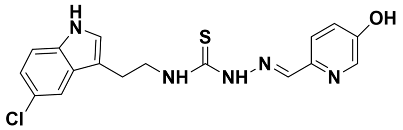	>64

617	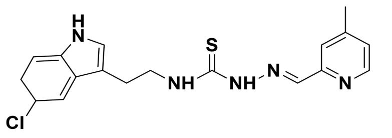	>64
501	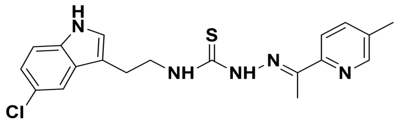	>64
598	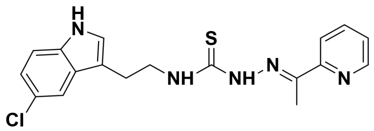	>64
1071	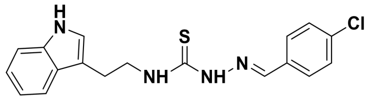	>64
696	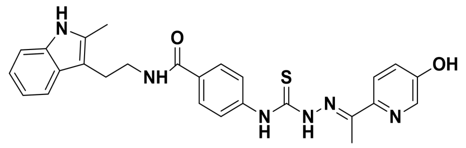	>64
700	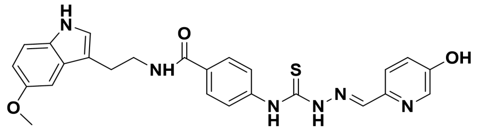	>64
1084	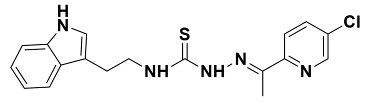	>64
639	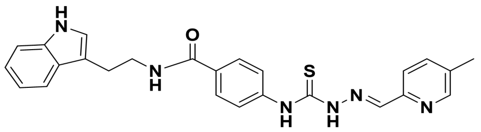	8
316	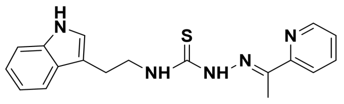	>64
1442	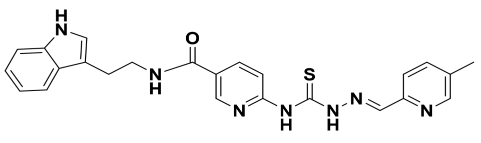	>64
1473	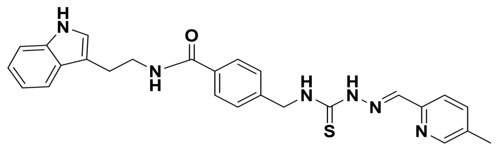	>64
746	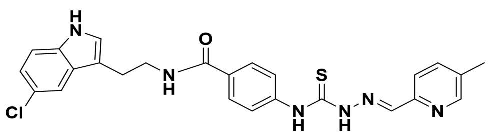	>64

690	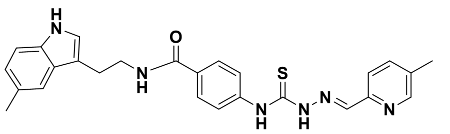	>64
699	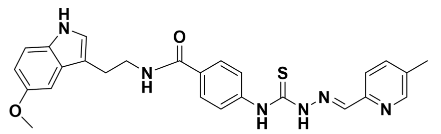	16
698	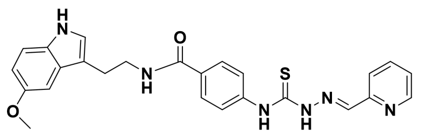	>64
695	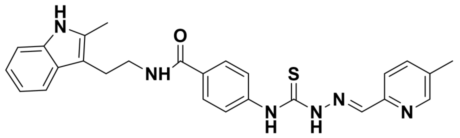	>64
1444	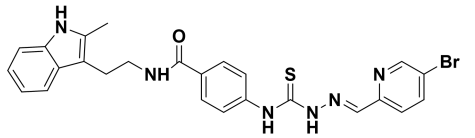	>64
1484	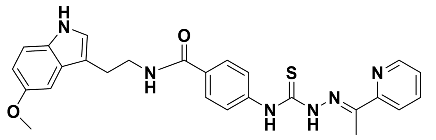	4
1425	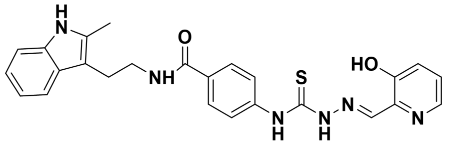	>64
1426	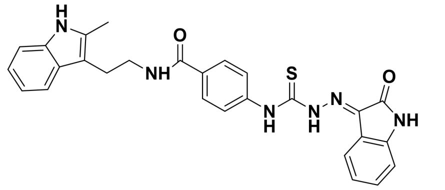	>64
1430	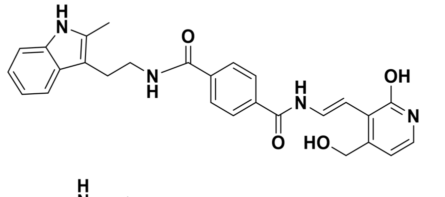	>64
1433	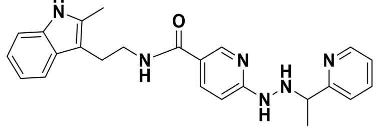	16

TABLE S2. MFCs of 19ak, 19k and 19t against *C. albicans* SC5314.

Compd.	MFC (μg/mL)
19ak	64
19k	>64
19t	64

TABLE S3. *In vitro* antifungal activities of 19ak against *C. albicans* SC5314 in the presence of H_2O_2.

Compd.	MIC (μg/mL)
19ak	0.125
19ak+1 mM H_2O_2	0.25
19ak+2 mM H_2O_2	0.5

TABLE S4. Primers used for qPCR.

Primers	Sequence (5' to 3')
NUO1-F	ACAGCTACTAATCAACCAGTGAG
NUO1-R	CAAGTGGGAATGATGCCGTAA
NUO2-F	AAGAAGCACAGAATTACAAGGACC
NUO2-R	CACAGATGCAGGCACAGACTT
MCI4-F	TTACAAACACCCAAATCCAAGACC
MCI4-R	CTTCAATCAAACCACCACCAATTTG
YMX6-F	ACCTTATCTGGTGTGGCATTAAC
YMX6-R	CTCCACGACATCTTCTAATCATTG
NDH51-F	ATTAAGAGGTCGTGGAGGTGC
NDH51-R	GCAGCAGTGGCATTCATAGC
RPL5-F	AACACAAGGCTAAATACAACTCACC
RPL5-R	CGTATCTTGGTAATTCGTGAGCAT
RPS3-F	TAGGTGAACAAGGTAGAAGAATCC
RPS3-R	CCAGAGATAACAACTTCAACACC
RPL3-F	AGAGAGAAGTTGTCGAAGCTG
RPL3-R	CTCAAGTGTTCAGCCCAGAC
RPS21-F	GCCAGTCAAGGAATACCAAATC
RPS21-R	CAGCTTTAATGGCAGAAGCAA
RPL14B-F	GCCAGAGTTACTGGTGGTATG
RPL14B-R	GATTGACCACCTGGACCTG
RPS12-F	AATTATTGGGTGAATGGGCTGG
RPS12-R	CAACAACACAAGAGGCACCA
CFL1-F	TGCAAGTAAATCCTCCAAGTCAAC
CFL1-R	CCTGCCCAATAACCAAGTAACC
FTR1-F	AGAAGCTGTTGTCTTCGTTGGTGG
FTR1-R	CAAATACCTCTGGAGAACAAACCA
FTH1-F	TGATGGCTCTTGTAAATGCTGC
FTH1-R	CCAGTCTCACTCACATCTAATCC

FTR2-R	GTTACAGCAGTTGACATGCCA
FTR2-F	TTGATCGCTGCTGGTTTGTTC
ZRT1-F	GCTTCTACAATTACCACACCACC
ZRT1-R	GGAACTGGCAAGATTGAACCT
ZRT2-F	ACTGGGCCAATAACAGAATACC
ZRT2-R	GGATATGGATTGGCATCAGCT
PRA1-F	TAGCTGAACATGCCAGGGACC
PRA1-R	CAATAACCAGCCCAGCCATC
CSR1-F	ACTTTCCCATTATGACGTTCACAC
CSR1-R	GCAAACCCAAAGTGAAGAAAGAGG
ACT1-F	TCCAGAAGCTTTGTTCAGAC
ACT1-R	TGCATACGTTCAGCAATACC

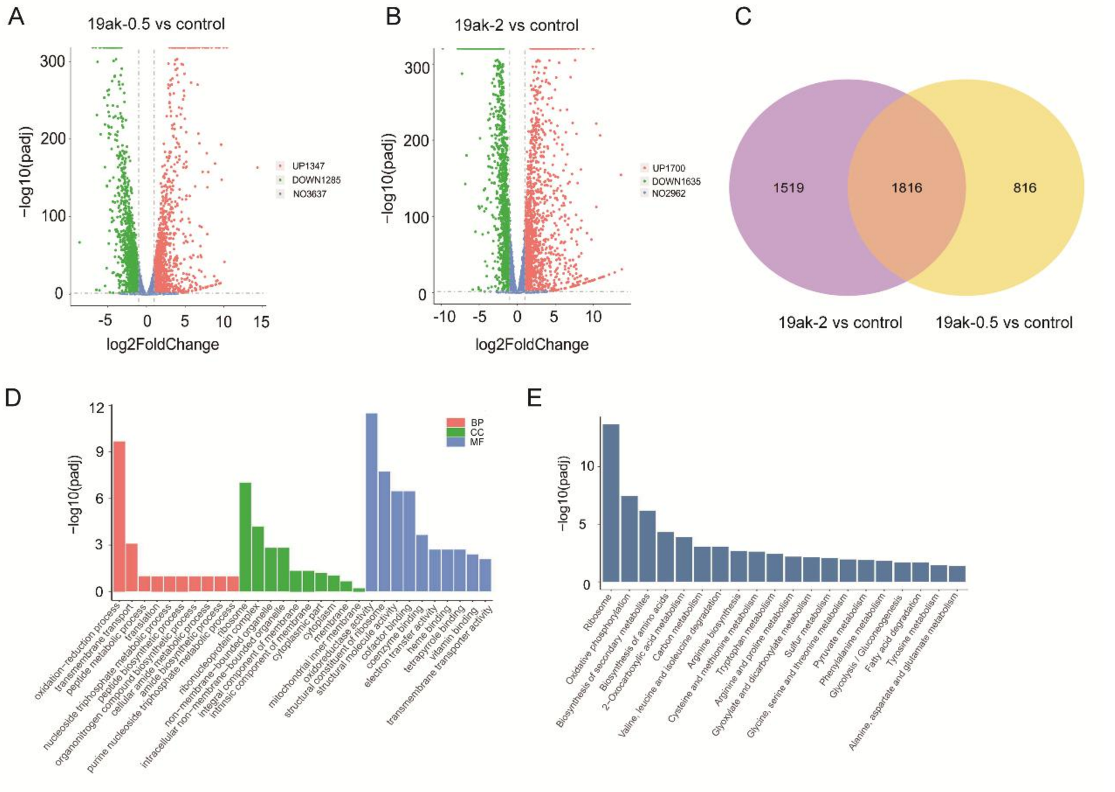

FIG S1 Differentially expressed genes (DEGs) in 19ak-treated *C. albicans* compared with control cells. *C. albicans* SC5314 cells were treated with 0.5 μg/mL or 2 μg/mL 19ak for 6 h and subjected to RNA-seq. (A, B) Volcano map of DEGs. In the sub-figure, the x-coordinate is the log2 fold change value, and the y-coordinate is -log10 (padj). Cells were treated with 0.5 μg/mL (A) or 2 μg/mL (B) 19ak and compared with the control. (C) Venn diagram of DEGs. (D) Gene Ontology (GO) annotations of regulatory DEGs in common between the groups treated with a high or low dose of 19ak. “BP”, “CC”, and “MF” in the sub-figure represent biological process, cellular component, and molecular function. The x-coordinate is the GO term of BP, CC, and MF, and the y-coordinate is -log10 (padj). (E) Annotation of DEGs using KEGG. The bottom x-axis indicates the main category, and the y-coordinate represents -log10 (padj).

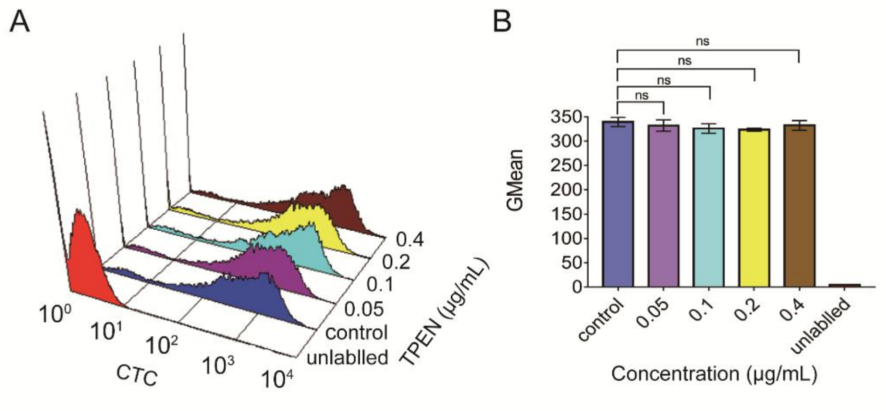

FIG S2 Effect of TPEN on the respiratory activity of *C. albicans* SC5314 cells were treated with TPEN (0.05, 0.1, 0.2, 0.4 μg/mL) for 6 h and stained with 1 mM CTC to measure respiratory activity. The fluorescence intensity was measured by flow cytometry (A), and GMean values were calculated (B). The MIC of TPEN against *C. albicans* SC5314 was 0.05 μg/mL.

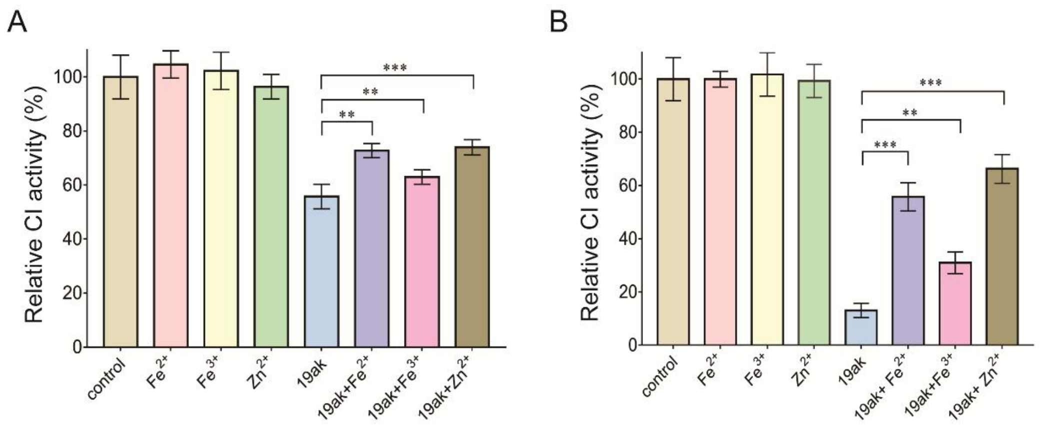

FIG S3 Effect of exogenous 100 μM metal ions on NADH dehydrogenase activity. Isolated mitochondria were treated with 0.125 μg/mL (A) or 1 μg/mL (B) 19ak for 6 h in the presence or absence of the indicated metal ions at a concentration of 100 μM. The activity of mitochondrial respiratory chain complex I was then measured.

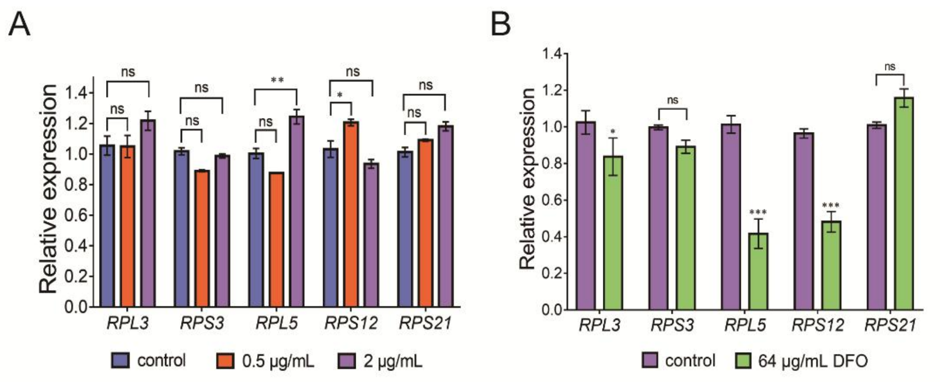

FIG S4 Effect of iron-specific chelators on ribosome biogenesis in *C. albicans*. (A) *C. albicans* SC5314 (1×10^6 cells/mL) were treated with 0.5 or 2 μg/mL CPX for 8 h at 30°C, followed by RNA extraction. The transcription levels of several genes associated with ribosome biogenesis were determined by qPCR. (B) *C. albicans* SC5314 was cultured in RPMI1640 medium and treated with 64 μg/mL DFO for 6 h at 30°C. The transcription levels of several genes associated with ribosome biogenesis were determined by qPCR. The bars represent means ± SD from three independent experiments. *$P < 0.05$, **$P < 0.01$, ***$P < 0.001$.

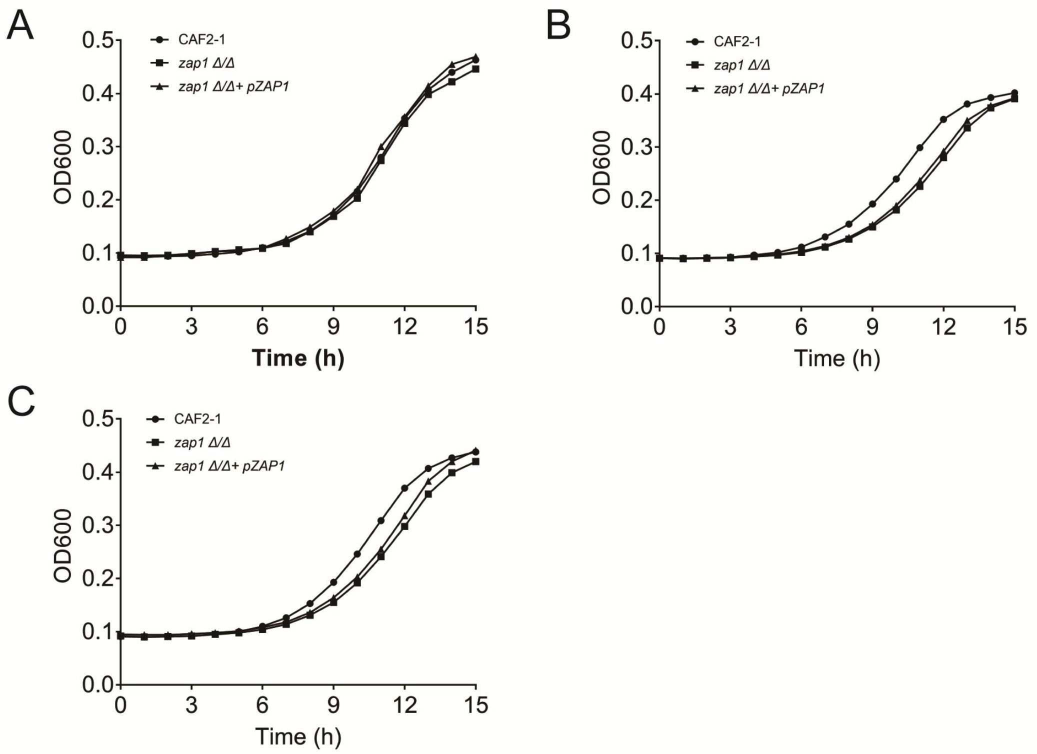

FIG S5 Effect of exogenous 100 μM Zn^{2+} on the sensitivity of the wild strain CAF2-1, the mutant strain *zap1Δ/Δ* and its complemented strain *zap1Δ/Δ*+ p*ZAP1*. (A) Cells that were not treated with 19ak as a control. (B) Cells treated with 0.25 μg/mL 19ak. (C) Cells treated with 0.5 μg/mL 19ak. Each point represents the mean of three replicates. The OD600 values at the final detection point were used to analyze significant differences.

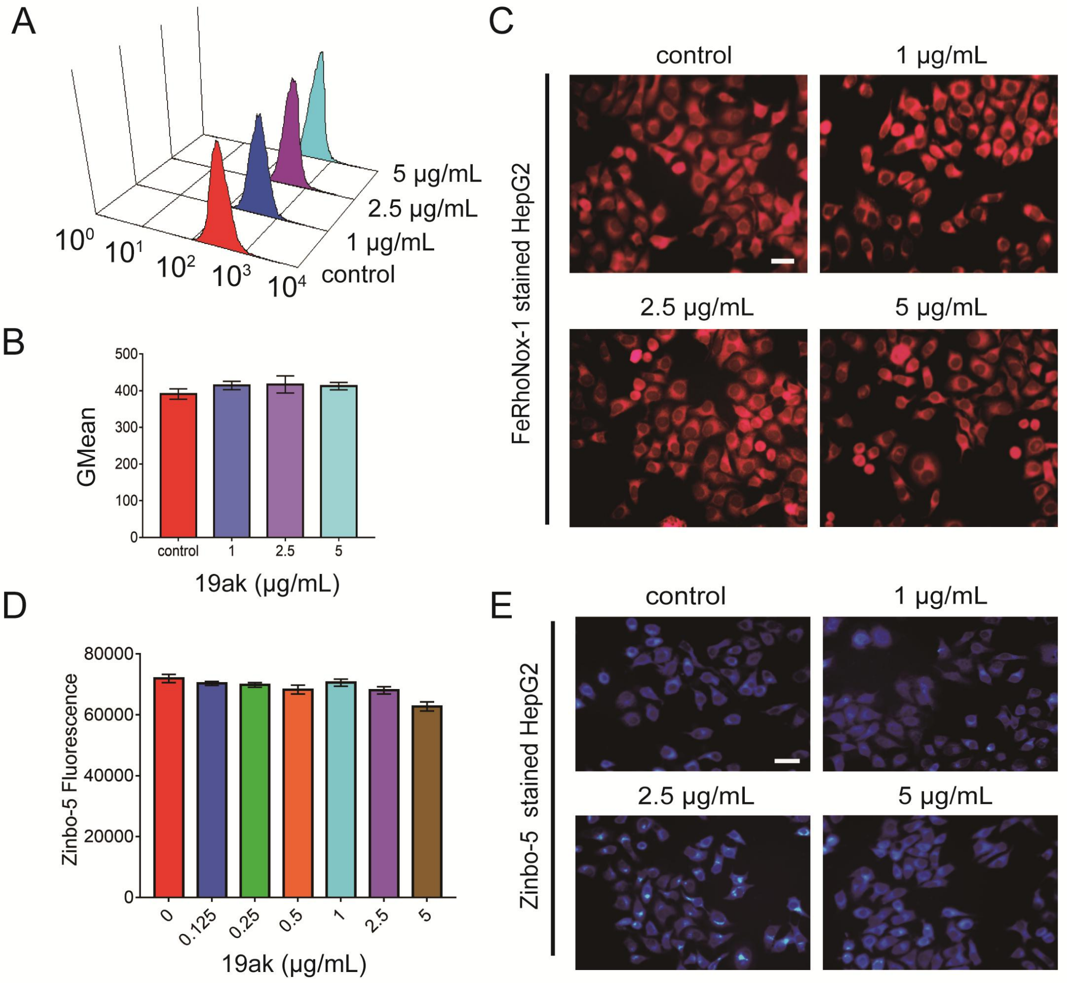

FIG S6 The effect of 19ak on metal chelation of mammalian HepG2 cells. (A-C) HepG2 cells were treated with 19ak for 12 h. The cells were then stained with FeRhonox-1 for measurement of fluorescence intensity by flow cytometry (A). The GMean value of fluorescence intensity was calculated (B). FeRhonox-1-stained cells were further observed by fluorescence microscopy (scale bars: 20 μm) (C). (D, E) HepG2 cells were treated with 19ak for 12 h. The cells were then stained with 10 μM Zinbo-5 and the fluorescence intensity was measured by a plate reader (D) or observed by fluorescence microscopy (E).

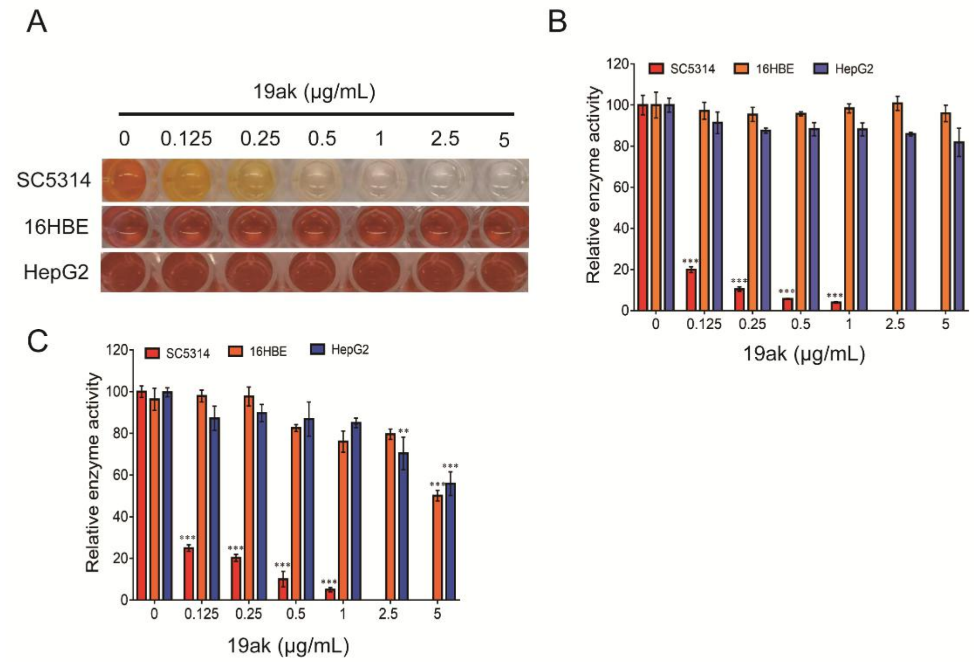

FIG S7 The effect of 19ak on mitochondrial dehydrogenase activity of mammalian cells. (A-C) *C. albicans* SC5314, mammalian cells of 16HBE or HepG2 were treated with 19ak (0, 0.125, 0.25, 0.5,1, 2.5, 5 μg/mL) for 12 h or 48 h. CCK-8 assays were performed to reveal the effects of 19ak on mitochondrial dehydrogenase activities of fungal cells or mammalian cells. The cell performance after 12 h of 19ak treatment were colored by CCK-8 solutions and imaged by a camera (A). The absorbance at 450 nm after 12 h (B) or 48 h (C) treatment was measured by a microplate reader. The bars represent means ± SD from three independent experiments. *$P < 0.05$, **$P < 0.01$, ***$P < 0.001$.

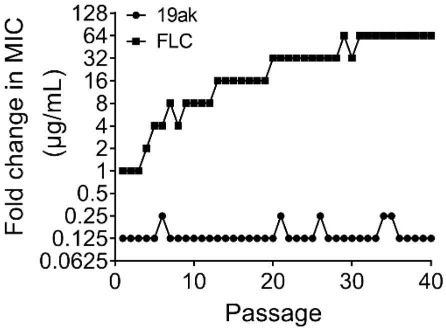

FIG S8 Evaluation of the potential of 19ak to induce the development of drug resistance. Serial passage experiments were performed by repeatedly exposing *C. albicans* SC5314 to 19ak or FLC. *C. albicans* cells cultured at 0.5 × MIC were removed and adjusted to 1 × 10^3 cells/mL using RPMI1640 medium for the next passage of MIC measurement. Each data point represents one passage.

SUPPLEMENTARY EXPERIMENTAL METHODS.

Compound library. The thiosemicarbazone derivatives Triapine and COTI-2 were purchased from Med Chem Express (MCE). The other 87 thiosemicarbazones used in this study were previously synthesized by our collaborators (1, 2). Their structures are shown in Table 1 and Table S1 to help the reader clearly understand the relationship between structure and activity.

Strains and culture conditions. Clinically derived *C. albicans* isolates, including G5, F5, and Gu5, were kindly provided by Professor Joachim Morschhäuser of the University of Würzburg. Other clinical *C. albicans* isolates were kindly donated by Professor Qingguo Qi of Shandong University and Qianfoshan Hospital of Shandong Province Other *Candida* strains were obtained from Jinan Central Hospital. The *zap1* mutant and its complemented strain were kindly provided by Professor Aaron P. Mitchell of Carnegie Mellon University (3). The *C. neoformans* strains used in this study was kindly donated by Professor Guojian Liao of Southwest University and *A. fumigatus* strain used in this study was provided by Professor Ling Lu of Nanjing Normal University. Before *in vitro* and *in vivo* experiments, all *C. albicans* strains were grown on YPD agar plates. After culturing at 30°C for 24 h, colonies of each strain were cultured in liquid YPD medium at 30°C with continuous shaking (200 rpm).

Antifungal susceptibility testing. To determine *in vitro* antifungal activities, the microbroth dilution method was carried out in RPMI1640 medium according to the guidelines of the Clinical and Laboratory Standards Institute (4, 5). The initial concentration in RPMI1640 medium was 1×10^3 CFU/mL for the *C. albicans* and *C. neoformans* suspensions but 1×10^4 CFU/mL for *A. fumigatus*. The range of final drug concentrations was 0.0156–64 μg/mL. The plates were incubated at 35°C for 24

h (for *C. albicans*, *C. krusei*, *C. tropicalis*, *C. parapsilosis*) or 48 h (for *C. neoformans, A. fumigatus*). The MIC was determined based on visible growth inhibition compared with the control. All experiments were performed in duplicate.

Cytotoxicity testing. The cell lines HK2 and 16HBE were used to evaluate the cytotoxicity of 19ak, 19k and 19t. Cells in DMEM containing 10% fetal bovine serum (Gibco) were seeded in 96-well tissue culture plates at a cell density of 1×10^4 cells/mL and incubated for 24 h to allow adhesion. The supernatant was then replaced with fresh DMEM containing various concentrations of 19ak, 19k and 19t. The plates were subsequently incubated for an additional 48 h at 37°C with 5% CO_2, followed by the addition of 10 μL of MTT solution to each well and incubation at 37°C for 4 h. The effects of the compounds on cell proliferation were detected by measuring the absorbance at 570 nm in a microplate reader (BioTek, VT, USA). Three independent experiments were conducted.

Rescue effect of exogenous metal ions on fungal growth inhibition by 19ak. Since the effects of thiosemicarbazone may be due to binding of intracellular metal ions, we investigated the impact of adding metal ions to the medium on the inhibitory effect of 19ak by performing antifungal susceptibility testing as described above in the presence of 50 μM, 100 μM, and 200 μM $ZnSO_4$, $FeSO_4$, and $FeCl_3$.

Gene Ontology (GO) and Kyoto Encyclopedia of Genes and Genomes (KEGG) enrichment analysis. The Cluster Profiler R package was used for Gene Ontology (GO) enrichment analysis of the DEGs, with *P*-value < 0.05 considered significantly enriched (6). We also analyzed KEGG pathways that were significantly enriched in DEGs with the aim of understanding the advanced functions and roles of biological systems based on large, high-throughput molecular data sets (7).

Analysis of respiratory activity. Biological reduction of CTC tetrazolium salt to

the fluorescent complex CTC-formaldehyde (CTF) is commonly used to indicate the intensity of microbial respiration. *C. albicans* SC5314 treated with 19ak was stained with 1 mM CTC for 30 min, followed by flow cytometry analysis of respiratory activity based on fluorescence intensity. Images of cells were obtained using a fluorescence microscope (IX71, Olympus, Tokyo, Japan). The Cell Counting Kit-8 (CCK-8) assay is used to measure the dehydrogenase activity in the mitochondria of *C. albicans* or mammalian cells. *C. albicans* SC5314 (5×10^6 cells/mL in RPMI1640 medium) were treated with different concentrations of 19ak in 96-well plates at 30°C for 12 h or 48 h. 10 μL of CCK-8 solution was added to each well. After an additional 2 h of incubation, OD450 in each well was detected with a microplate reader. For mammalian cells, cell suspensions (1×10^4 cells/mL) in DMEM medium contained 10% FBS are distributed in 96-well plates and placed in an incubator (37°C, 5% CO_2). After incubation with various concentrations of 19ak for 12 h or 48 h, a CCK-8 reduction assay was performed to determine the mitochondria dehydrogenase activity of mammalian cells.

Mitochondrial isolation and detection of mitochondrial respiratory chain complex activity. Mitochondria were isolated from *C. albicans* spheroplasts as previously described (8). A dilution series of 19ak was added to the mitochondrial suspensions to obtain final concentrations of 0, 0.125, 0.25, 0.5, and 1 μg/mL and incubated for 6 h at 30°C. The activities of mitochondrial respiratory chain complexes I-IV were determined by using the Micro Mitochondrial Respiratory Chain Complex Activity Assay Kit (Solarbio, Beijing, China). The concentration of protein was determined using the BCA Protein Assay Kit (Beyotime, Haimen, China). Mitochondrial complex activity was expressed as nmol/min/mg protein.

Analysis of mt$\Delta\psi$. Rh123 is a fluorescent stain that responds directly to mt$\Delta\psi$. *C.*

albicans SC5314 cells (1 × 10^6 cells/mL) were challenged with various doses of 19ak and incubated at 30°C for 6 h, followed by staining with 20 μM Rh123 for 30 min in the dark and detection by flow cytometry (Becton Dickinson, CA, USA).

Measurement of intracellular ATP production. To explore whether 19ak affects intracellular ATP content by damaging mitochondria, we treated *C. albicans* SC5314 cells (1 × 10^6 cells/mL) with different doses of 19ak. After lysing the cells in lysis buffer, ATP levels were measured with the ATP Assay Kit (Beyotime, Haimen, China) according to the manufacturer's instructions. Cellular protein concentrations were determined using a BCA Protein Assay Kit (Beyotime, Haimen, China). The intracellular ATP content was calculated by referring to a standard curve and normalized using the protein content of each sample for expression as nmol/mg protein.

ROS production assay. Logarithmic-phase *C. albicans* SC5314 cells (1 × 10^6 cells/mL in RPMI1640 medium) were incubated in the presence or absence of 19ak for 6 h, followed by incubation with 2′,7′-dichlorofluorescein diacetate (DCFH-DA) in the dark for 30 min. The fluorescence intensity was then measured by flow cytometry, and the data were analyzed with Win MDI 2.9 software.

Influence of oxidant or antioxidant molecules on 19ak activity. The MIC of 19ak in the presence of 1–2 mM H_2O_2 was measured using the broth microdilution assay as described above. The effects of NAC, Tu and DTT on the growth of 19ak treated fungal cells were measured using a time-growth curve. *C. albicans* SC5314 cells were adjusted to 1 × 10^5 cells/mL in SC medium containing 5 mM NAC, 5 mM Tu, or 0.5 mM DTT. Various concentrations of 19ak were added to the cultures and incubated at 30°C for 15 h. A microplate reader was used to measure the absorbance at 600 nm, and the growth curves were plotted.

Determination of SOD activity. SOD activity was measured using the Superoxide Dismutase Activity Assay Kit (Njjcbio, Nanjing, China) following the manufacturer's instructions. An overnight culture of *C. albicans* SC5314 was adjusted to 1 × 10^7 cells/mL with RPMI1640 medium and treated with 19ak at a final concentration of 0, 0.125, 0.25, 0.5, or 1 μg/mL for 6 h. After collection by centrifugation, the cells were broken by glass beads in extraction buffer and centrifuged at 4°C. The absorbance of the supernatant of each sample was measured with a microplate reader at 450 nm.

Determination of catalase (CAT) activity. CAT activity was measured by using a Catalase Assay Kit (Solarbio, Beijing, China) following the manufacturer's instructions. An overnight culture of *C. albicans* SC5314 was adjusted to 1 × 10^6 cells/mL with RPMI1640 medium and treated as described above for SOD activity. The decomposition of H_2O_2 is directly accompanied by a decrease in absorbance at 240 nm, and the difference in absorbance per unit time (ΔA240) is a measure of catalase activity.

Quantitative real-time polymerase chain reaction (qPCR) analysis. *C. albicans* SC5314 cells (1 × 10^6 cells/mL) were incubated with or without 19ak (0.5, 2 μg/mL) in RPMI1640 medium for 6 h. Total RNA was extracted using the hot phenol method, and the RNA quality was analyzed using a spectrophotometer (Eppendorf, Hamburg, Germany). The RNA was converted to cDNA using a cDNA Reverse Transcription kit (Accurate Biotechnology, Hunan, China). PCR detection based on SYBR Green was performed on an Eppendorf real-time PCR system (Eppendorf, Hamburg, Germany). The primer sequences used in this study are shown in Table S4.

Drug resistance study. Drug resistance studies were carried out by repeatedly treating *C. albicans* with an antifungal agent followed by MIC determination using

the broth microdilution assay (9). Specifically, *C. albicans* cells incubated at 0.5 × MIC were adjusted to 1×10^3 cells/mL using RPMI1640 medium for the next passage of MIC measurement. This process was repeated 40 times.

Statistical analysis. One-way ANOVA test or Student's t-test (two-tailed, unequal variance) was used to assess the statistical significance of the difference between treatment group and control group. The Mantel-Cox test was used to compare differences between groups in survival analysis. Data were showed as mean ± standard deviation (SD). Statistical significance was determined according to the P value. $*P < 0.05$, $**P < 0.01$, $***P < 0.001$.

Reference

1. Zhao B, Zhang X, Yu T, Liu Y, Zhang X, Yao Y, Feng X, Liu H, Yu D, Ma L, Qin S. 2021. Discovery of thiosemicarbazone derivatives as effective New Delhi metallo-β-lactamase-1 (NDM-1) inhibitors against NDM-1 producing clinical isolates. Acta Pharm Sin B 11:203-221.

2. He Z, Qiao H, Yang F, Zhou W, Gong Y, Zhang X, Wang H, Zhao B, Ma L, Liu HM, Zhao W. 2019. Novel thiosemicarbazone derivatives containing indole fragment as potent and selective anticancer agent. Eur J Med Chem 184:111764.

3. Nobile CJ, Nett JE, Hernday AD, Homann OR, Deneault JS, Nantel A, Andes DR, Johnson AD, Mitchell AP. 2009. Biofilm matrix regulation by *Candida albicans* Zap1. PLoS Biol 7:e1000133.

4. Clinical and Laboratory Standards Institute. Reference method for broth dilution antifungal susceptibility testing of yeasts-Third Edition: Approved Standard M27-A3. CLSI, Wayne, PA, USA; 2008.

5. Clinical and Laboratory Standards Institute. Reference method for broth

dilution antifungal susceptibility testing of filamentous fungi-Second Edition: Approved Standard M38-A2. CLSI, Wayne, PA, USA; 2008.

6. Young MD, Wakefield MJ, Smyth GK, Oshlack A. 2010. Gene ontology analysis for RNA-seq: accounting for selection bias. Genome Biol 11:R14.

7. Ogata H, Goto S, Sato K, Fujibuchi W, Bono H, Kanehisa M. 1999. KEGG: Kyoto Encyclopedia of Genes and Genomes. Nucleic Acids Res 27:29-34.

8. Helmerhorst EJ, Murphy MP, Troxler RF, Oppenheim FG. 2002. Characterization of the mitochondrial respiratory pathways in *Candida albicans*. Biochim Biophys Acta 1556:73-80.

9. Chang W, Liu J, Zhang M, Shi H, Zheng S, Jin X, Gao Y, Wang S, Ji A, Lou H. 2018. Efflux pump-mediated resistance to antifungal compounds can be prevented by conjugation with triphenylphosphonium cation. Nat Commun 9:5102.
